# UV Filters: Challenges and Prospects

**DOI:** 10.3390/ph15030263

**Published:** 2022-02-22

**Authors:** Ana Jesus, Emília Sousa, Maria T. Cruz, Honorina Cidade, José M. Sousa Lobo, Isabel F. Almeida

**Affiliations:** 1 UCIBIO—Applied Molecular Biosciences Unit, MedTech, Laboratory of Pharmaceutical Technology, Department of Drug Sciences, Faculty of Pharmacy, University of Porto, 4050-313 Porto, Portugal; anaaimjesus@gmail.com (A.J.); slobo@ff.up.pt (J.M.S.L.); 2 Associate Laboratory i4HB—Institute for Health and Bioeconomy, Faculty of Pharmacy, University of Porto, 4050-313 Porto, Portugal; 3 Laboratory of Organic and Pharmaceutical Chemistry, Department of Chemical Sciences, Faculty of Pharmacy, University of Porto, 4050-313 Porto, Portugal; esousa@ff.up.pt; 4 CIIMAR—Interdisciplinary Centre of Marine and Environmental Research, Avenida General Norton de Matos, S/N, 4450-208 Matosinhos, Portugal; 5 Faculty of Pharmacy, University of Coimbra, 3004-531 Coimbra, Portugal; trosete@ff.uc.pt; 6 Center for Neuroscience and Cell Biology, 3004-504 Coimbra, Portugal

**Keywords:** UV filters, challenges, toxicity, photostability, prospects, natural products, synthetic derivatives

## Abstract

The use of sunscreens is an established and recommended practice to protect skin from solar-induced damage. Around 30 UV filters can be used in sunscreen products in the European Union, which ought to follow the requirements of the regulation 1223/2009 to ensure their efficacy and safety for humans. Nevertheless, low photostability and putative toxicity for humans and environment have been reported for some UV filters. Particularly, the negative impact in marine organisms has recently raised concern on the scientific community. Therefore, it is important to develop new UV filters with improved safety profile and photostability. Over the last two decades, nearly 200 new compounds have revealed promising photoprotection properties. The explored compounds were obtained through different approaches, including exploration of natural sources, synthetic pathways, and nanotechnology. Almost 50 natural products and around 140 synthetic derivatives, such as benzimidazoles, benzotriazoles, hydroxycinnamic acids, xanthones, triazines, among others, have been studied aiming the discovery of novel, effective, and safer future photoprotective agents. Herein, we provide the reader with an overview about UV filters’ challenges and prospects, offering a forward-looking to the next-generation of UV filters.

## 1. Introduction

Sunlight has several beneficial effects in human, such as the production of vitamin D, and induction of β-endorphin expression, which improve well-being [1]. However, excessive sunlight exposure is responsible for photo-induced skin damage, namely solar sunburn, hyperpigmentation, photoaging, skin photosensitisation, and skin cancer [2], when protective measures, namely the use of sunscreen and the use of adequate clothes and accessories, are not adopted [3]. Photoprotective measures are more ancient than the first sunscreen’s appearance in the 1900s, and ancient civilisations used plant extracts to protect their skin from sunburns for a long time [4]. Many of the chemical compounds present in natural extracts with photoprotective properties are now part of sunscreens [5]. In fact, most of UV filters are inspired in natural products, specifically of botanical, animal, or mineral origin [5]. In 1928, benzyl salicylate was discovery for its photoprotective action against UVB radiation, but it was only commercialised in 1935 in the first sunscreen “Ambre Solaire” [6,7]. Later, almost 50 years, avobenzone and its derivatives appeared as the first UV filters that ensure protection against UVA radiation [8]. Currently, in Europe, there are a total of 29 approved UV filters [9] (compounds **1**–**35** (Table S1)), complying with the regulations that ensure their effectiveness and safety for humans. UV filters can be classified concerning their ability to absorb the UV radiation (UVR), as UVA, UVB or broad-spectrum UV filters (UVA and UVB) [10]. Additionally, these products can also be branched into organic or inorganic, where organic filters are only capable to absorb the UVR, while inorganic filters can reflect and scatter the UVR [11]. In recent decades, the safety of UV filters for humans and environment has been called into question. In fact, many studies have confirmed the detection of UV filters in human biological samples [12,13] and in marine organisms [14,15,16,17], thus confirming the hypothesis of UV filter-derived toxic effects. The presence of particular chemical moieties in UV filter structures confers intrinsic toxicity [18,19]. Photoinstability occurs for UV filters that photoisomerise or photodegrade, and consequently can generate toxic photodegradation products and loss of photoprotective action [20]. Therefore, over the last two decades, new natural products from botanical and marine sources [21,22,23] and synthetic derivatives [21,24,25,26] have been investigated, along with the use of nanotechnology approaches [11] as strategies to find new, more effective, safer and more stable UV filters. With this, our aim is providing the reader with a comprehensive review about the studies available in the literature focused on the main challenges and the future perspectives of UV filters.

## 2. Challenges

The main challenges associated with the UV filters present in sunscreens are their photoinstability, environmental impact, and human toxicity. As mentioned before, these are some points to consider in the development of new effective and safer photoprotective agents.

### 2.1. Photostability

The stability of sunscreens is an essential requisite to ensure the photoprotection and safety. UV filters absorb UVR and enter in excited energetic levels [27]. Then, the energy is released, and the chemical molecule returns to its initial energetic level. During this process, some UV filters undergo photoisomerisation and even irreversible cleavage of bonds [28]. The mechanisms associated with this phenomenon include the formation of photodegradation products, which can negatively influence the sunscreen’s effectiveness. These degraded derivatives could also be toxic due to interaction with the constituents present in cells and/or damage the DNA. Moreover, they can also affect the stability of the other ingredients present in the formulation [27,29]. 

**Figure 1:**
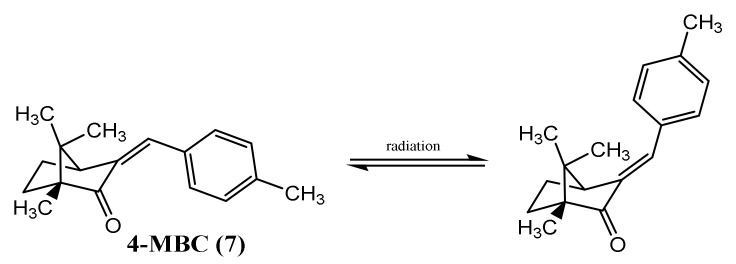
Interconversion between E and Z isomers when 3-(4-methylbenzylidene) camphor (**4-MBC**) (**7**) is exposed to UV radiation.

The UV filter 3-(4-methylbenzylidene) camphor (4-MBC) (**7**) (Figure 1) suffers *E*-*Z* photoisomerisation. Nevertheless, 4-MBC (**7**) is considered stable due to the similarity of the UV spectra before and after irradiation, as well as the minimum change of the photoprotective action, before and after the irradiation [30]. 

**Figure 2:**
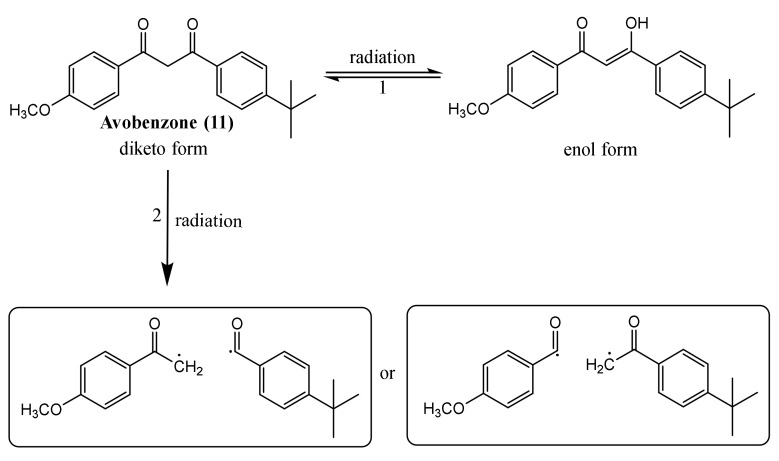
Mechanisms that could be triggered when avobenzone (**11**) is exposed to UV radiation, (1) photoisomerisation between di-keto and enol tautomers, or/and (2) formation of two radicals.

In 1995, Schwack and Rudolph reported insights that contributed to understand the photostability of avobenzone (**11**), a widely used UVA filter. The results showed the presence of around 10 photodegradation products, after 8 h of UV exposure with wavelengths of 260 or 320 nm [31]. It was verified that UV irradiation can promote the keto-enol isomerisation of **11** (Figure 2 (1)), and the fragmentation into two radicals (Figure 2 (2)), which after could form a phenacyl and benzoyl radicals that could generate reactive by-products, through reaction with others UV filters or fragments [28,30]. 

**Figure 3:**
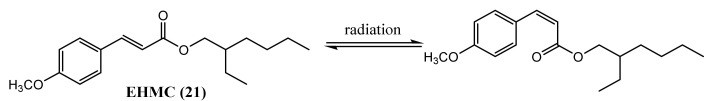
Interconversion between E and Z isomers when EHMC (**21**) is exposed to UV radiation.

Similarly to 4-MBC (**7**), ethylhexyl methoxycinnamate (EHMC) (**21**) was also studied regarding to this topic. In 1999, Tarras-Wahlberg et al. verified by high-performance liquid chromatography (HPLC) analysis that samples of **21** previously irradiated with UVR (λ = 290–320 nm) showed the presence of an additional band associated with the *Z* isomer of the UV filter, confirming the conversion between *E* and *Z* isomers after UV exposure (Figure 3) [28,30]. 

The photodegradation of 2-ethylhexyl-4-dimethylaminobenzoate (Padimate O) (**24**) was also evaluated confirming its photoinstability for UVA and photostability for UVB radiation. This compound tends to promptly decompose after irradiation, forming two photoproducts, one as a result of the loss of methyl group from the initial molecule, and the other product with an extra value of atomic number, probably associated with the oxidation of the amine group, directly linked to the aromatic ring (Table 1, 2nd entry) [30]. It is noteworthy to mention that, recently, it was reported the conversion of octocrylene (**30**) into a benzophenone derivative through a retro-aldol condensation reaction, which increases the phototoxic potential of this UV filter [32]. 

In Table 1, the photodegradation products of the UV filters reported above are presented. Photodegradation products of avobenzone (**11**) and 2-ethylhexyl-4-dimethylaminobenzoate (**24**) were detected by analytic chromatographic techniques, such as gas chromatography-mass spectrometry (GC-MS) or HPLC. The number of photodegradation products varied according to the solvent used, time of exposure, and dose of radiation. The time of exposure to the radiation used ranged from 20 min to 140 h, and the solvents used for the photolysis studies reported here were water, ethyl acetate, dimethylsulfoxide (DMSO), and cyclohexane. UV filter avobenzone (**11**) is one of the photoprotective ingredients that shows the highest number of photodegradation products. Three photodegradation products were detected when this UV filter was exposed to the highest time of exposure (100 h) in cyclohexane [33]. The number of photodegraded products increased 4 times when the dose of radiation augmented to 692 J/cm^2^, maintaining the solvent cyclohexane and decreasing the time of exposure to 8 h [31]. This phenomenon could be a result of a high dose of radiation used that contributed to the formation of radical species of the parent UV filter and induced the formation of new compounds derived from the reaction between the different radicals formed [33]. Using the same dose of radiation (100 J/cm^2^), but different time of exposition, type of radiation exposure and solvent, Padimate O (**24**) could present different photo-induced degradation products; however, all of them showed as susceptible point the aromatic amine group [30,33]. 

#### Strategies to Improve the Photostability of Organic UV Filters

Several approaches could be used in order to ameliorate the photostability of photoprotective agents, such as the introduction of antioxidants [29], encapsulation [34], multiple association of UV filters [35,36,37,38], and the addition of quenching molecules [39] in the sunscreen’s formulation [40]. 

Afonso et al. (2014) reported the stabilising effect of antioxidants, namely vitamin C, vitamin E, and ubiquinone, when used concomitantly in the formulation, which improved the photostability of avobenzone (**11**) with antioxidant concentrations of 10 µg/mL, 2.5 µg/mL, and 2.5 µg/mL, respectively, as well as the improvement of the SPF value of the formulation with ubiquinone [29]. In addition, it was also noticed that *trans*-resveratrol combined with β-carotene ameliorates the photostability of the UV filters, in particular of avobenzone (**11**) [37]. Duarte et al. (2019) tested the micro-encapsulation of EHMC (**21**) in alginate microparticles and studied its stabilisation effect in combination with vitamin E, confirming an improved capacity of stabilisation, as a result of the decrease in the photoisomerisation of the UV filter [34]. Additionally, gelatine micro- and nano-spheres improved skin compatibility, safety, and efficacy [41,42,43] and alginate microparticles influenced photostability, through the reduction of the EHMC’s (**21**) photoisomerisation [34]. Al-Rawashdeh et al. (2010) reported the micro-encapsulation of commercial UV filters (oxybenzone (**10**), EHMC (**21**), and octocrylene (**30**)) in hydroxypropyl-β-cyclodextrins system that successfully acted as photostabilisers [44]. Studies with cyclodextrins encapsulation using other UV filters have been also reported [45,46,47]. Moreover, the stabilisation of avobenzone (**11**) mediated by the combination with other UV filters, such methylbenzylidene camphor (**7**) or octocrylene (**30**) [48], or with bis-ethylhexyloxyphenol methoxyphenyl triazine (**27**) [35], was proved as an advantageous option that confers more stability to the photounstable UV filters. Quenchers could be used as a stabilisation strategy for some photounstable UV filters and can be related to the excited energetic state where the molecule migrates after receiving and absorbing UVR. Accordingly to this, they can be classified as singlet–singlet, triplet–triplet, and/or reactive oxygen species (ROS) quenchers [49]. For instance, Paris et al. (2019) prepared a methylated derivative of avobenzone (**11**) in order to block the conversion between keto-enol forms, and a new triazine triplet quencher molecule was added, reducing the undesirable effects of the UV filter by successful quenching the triplet excited state of UV filter [39].

**Table 1:** Photodegradation products of commercialised UV filters that were study regarding to their photo-induced degradation.

UV Filter	Conditions	Photodegradation Products	Reference
Avobenzone (**11**)	Time: 100 h Lamp: mercury vapour immersion Dose: 100 J/cm^2^ Solvent: cyclohexane	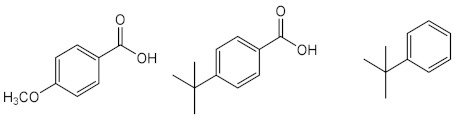	[33]
	Time: 8 h Lamp: SOL 500 Dose: 692 J/cm^2^ Solvent: ethyl acetate	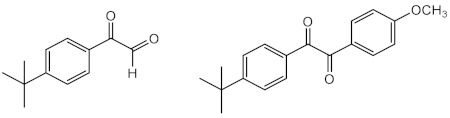	[31]
	Time: 8 h Lamp: SOL 500 Dose: 692 J/cm^2^ Solvent: cyclohexane	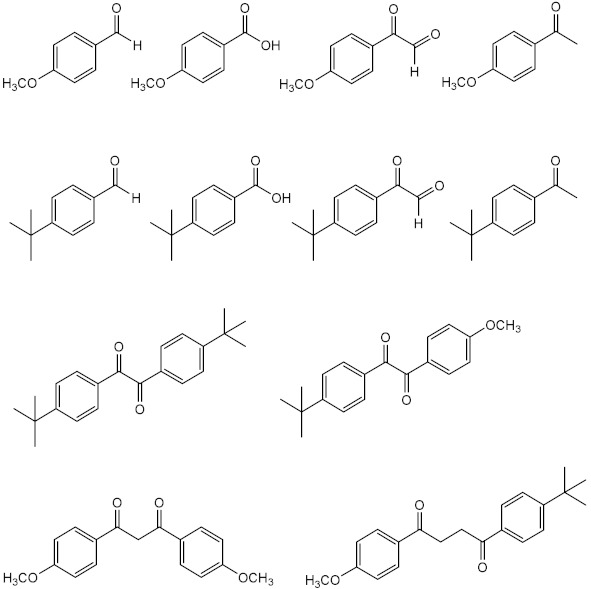	
	Time: 8 h Lamp: SOL 500 Dose: 692 J/cm^2^ Solvent: dimethylsulfoxide (DMSO)	Did not occurred degradation but the UV filter photoisomerised	
	Time: 2 h Lamp: Xenon Dose: 60 kJ/m^2^ Solvent: water	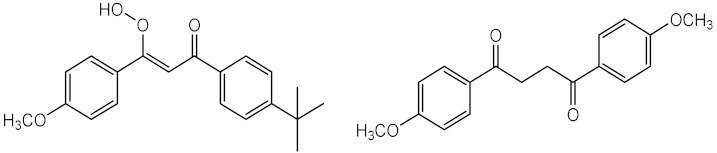	[50]
2-ethylhexyl-4-dimethylaminobenzoate (Padimate O) (**24**)	Time: 20 min Lamp: UVASUN 2000 Dose: 100 J/cm^2^ Solvent: petroleum jelly	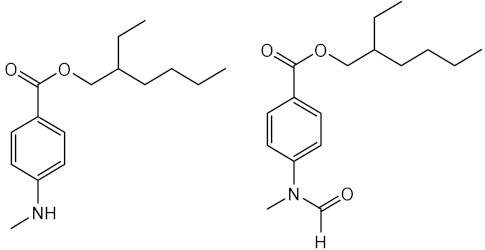	[30]
	Time: 140 h Lamp: mercury vapour immersion Dose: 100 J/cm^2^ Solvent: cyclohexane	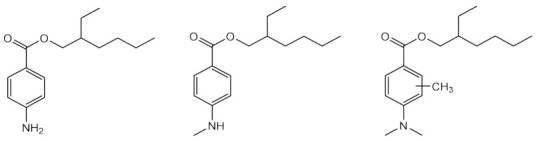	[33]

### 2.2. Toxicity (Human and Environmental)

**Figure 4:**
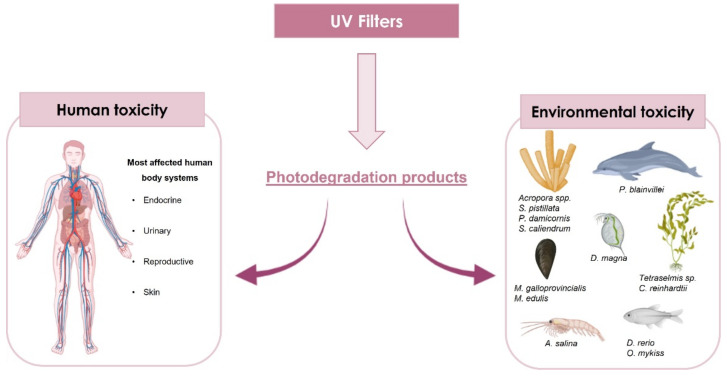
Resume of the main systems affected by UV filter’s toxicity.

The concern about the negative impact of UV filters on environment and organisms is growing day-by-day. The research community is trying to find alternatives and solutions to minimise the risks posed by these actual emergent pollutants [51]. The impact that sunscreen agents could have in organisms is relevant, namely in marine organisms. Recently, several studies revealed the dangerous and noxious effects of UV filters towards diverse aquatic species, such as mussels [14,52,53], algae [54], crustaceans [55], corals [56,57,58,59], sea urchins [16], fish [60,61,62], and even in dolphins [63]. There are two ways of studying the toxicological effects in marine organisms: by determining the concentration of UV filters in a specific organism, by collecting the marine organism in the environment, or through the organism’s exposure to a specific range of UV filters’ concentrations and subsequently verifying the effects [17]. In the next sections, the possible effects that UV filters may have in human beings and in the environment, namely in marine organisms, will be described. Figure 4 depicts the main human systems and marine organisms that suffer the negative impact of the UV filter’s toxicity.

#### 2.2.1. Human Safety

There are few studies about the toxicity for human beings. Some studies reported the ability of UV filters to penetrate the skin and reach the blood circulation, triggering concerns about the possible negative impact of UV filters in human body. Previous studies revealed that the size of the particles is a crucial parameter to consider. For instance, nanoparticles with smaller sizes could lead to a reactive response by inducing cytotoxicity [64]. Recently, it was reported that nanoparticles remain in the *stratum corneum* and do not penetrate into the skin [65]. Regarding organic UV filters, some reports evidenced their presence in biological samples, such as in urine and blood samples, particularly benzophenone and cinnamate derivatives [66]; however, some strategies could be applied as a preventive measure to avoid the systemic effect, namely the use of silicon spheres [67], or mesoporous silica [68]. 

The Organisation for Economic Co-operation and Development (OECD) published a document with standardised tests to evaluate the potential endocrine disruption molecules [69]. In accordance, the potential endocrine disruption of UV filters in biological samples, namely in placenta and human sperm have been already reported. Witorsch et al. (2010) referred the negative impact of some classes of compounds, including UV filters that crossed the skin layers; nevertheless, this study highlighted that serum and reproductive hormones levels were not affected by this exposure [70]. Lately in 2016, Rehfeld et al. studied the in vitro effect of UV filters in men’s fertility, by hypothesising the possibility of UV filters mimicking progesterone, considering that both hormone and UV filter can interfere with the Ca^2+^ channel signalling and, consequently, activate certain biological processes [71]. In total, 10 of the 29 UV filters analysed (concentration 10 µM) induced Ca^2+^ production, and other nine activated the respective Ca^2+^ channel. In addition, benzylidene camphor sulfonic acid (**5**) and 3-benzylidene camphor (**7**) inhibited progesterone, and consequently induced the Ca^2+^ channel pathway, which leads to men’s infertility [71]. These results were performed using in vitro assays and human in vivo studies are needed to validate the effect of UV filters in men’s fertility. Indeed, in 2018, a study addressed the clinical impact of 29 UV filters on the progesterone present in human sperm, concluding that the exposure to these compounds may decrease the men’s fertility, although more research studies are required to confirm these in vivo results [13]. 

Valle-Sistac et al. (2016) also investigated the possible endocrine disrupting effects resulting from UV filters exposure in human embryos [12]. The research study was performed with placentas of volunteers exposed to some UV filters. The frequency of detection of UV filters (benzophenone derivatives) varied between 17% and 100%. Of all the benzophenone-type UV filters tested, benzophenone-4 (BP-4) (**12**) was the UV filter that tends to accumulate more among this type of compounds in placenta with concentrations between 0.25 ng/g and 5.41 ng/g [12].

Several research groups have reported other negative effects of UV filters using in vivo models, such as rats [72,73], insects [74], fish [75,76], among others. Estrogenic, androgenic, and thyroid activities are included in the endocrine disrupting effects of UV filters [77], but other inherent effects have been reported, namely cytotoxicity [78], behavioural changes [79], and neurotoxicity [80]. Nevertheless, the use of human biological samples is always required to confirm the repercussion of UV filters in human beings. Possibly, the most toxic UV filters are mentioned to be the benzophenone and dibenzoylmethane derivatives due to the presence of aromatic ketones, which are not easily recognised by metabolic enzymes in humans, thus inducing toxic and allergic reactions [18], and also because of the photoisomerisation process leading to the formation of toxic and reactive photodegradation products [28]. Interestingly, through the analysis of the UV filters’ chemical structure it is possible to detect the presence of some similar points in chemical structure of others UV filters already reported by their toxic effects, namely cinnamates, octocrylene (**30**), and camphor derivatives; for instance, the double bond of the α,β-unsaturated system presents in both UV filters is known for their susceptibility to suffer Michael addition reactions with the skin proteins, forming protein adducts, that can lead to skin sensitisation reactions and to allergic contact dermatitis [19]. Sulphonated compounds have been reported for accumulate and generate toxicity in marine/aquatic systems and organisms [81]. In addition to environmental toxicity, the sulfonate group’s ability to act as DNA alkylating agents, and as a genotoxic agent in bacterial and mammalian cells was already reported [82]. 

#### 2.2.2. Environmental Safety

The occurrence of UV filters in different locations worldwide with concentration values between ng/L and µg/L has been studied in lakes, seawaters, sediments, rivers, estuaries, and in aquatic organisms [83]. In fact, the harmful effects of UV radiation leaded to an extensive production and use of photoprotective products during vacancies, which resulted in an increase in UV filters present in an environment, namely in aquatic ecosystems [84]. One study reported the presence of a UV filter EHMC (**21**) and other contaminants in freshwater fish of four Spanish rivers, with a concentration of 242 ng/g [85]. It is noteworthy mentioning that the presence of these compounds in marine waters, will after some time deposit in sediments, contributing for the bioaccumulation in both marine and terrestrial organisms, through absorption, abiotic and biotic degradation, hydrolysis, and photolysis [84,86,87]. The contamination of terrestrial environment could also occurred by wastewaters discharges, disposal of product packages in inappropriate locations, and even in indoor dust that drives to an environment issue, contaminating both land and terrestrial organisms [84]. Bioaccumulation and toxicological effects are the main issues associated with marine contamination by UV filters that could induce the persistence of these emergent contaminants through the food chain [84]. Considering the relevance of this topic, herein some studies reporting the bioaccumulation and negative impact of UV filters in marine organisms are discussed. 

##### Corals

Since the 1990s, the effects of UVR on marine organisms started to be investigated, especially in coral reefs. Shick et al. (1996), reported some of the negative effects of UVR on coral reefs, showing the sensitivity of corals to excessive UVR exposure. These radiations also affect endosymbiotic organisms, namely dinoflagellates organisms (*zooxanthellae*) that live in coral reefs depending on the coral species, the ambient, and the exposure to UVR [57]. Moreover, the chronic exposure of UVR on coral reefs could lead to irreversible effects, such as inhibition of cell growth and bleaching, one of the most visible effects. The negative impact on photosynthesis, cell division of endosymbiotic organisms, reproduction, and induction of photo-oxidative stress are also other effects that UVR can cause on corals [57]. Nonetheless, changes in coral’s behaviour, such the relocation of the coral reefs and the production of melanin, fluorescence pigments and mycosporine-like amino acids (MAAs), were some of the photoprotective measures adopted by coral for self-defence from excessive UVR [57]. Following this study, samples of corals collected in Pearl River Estuary of South China Sea demonstrated the presence of the UV filter BP-3 (**10**) in all the coral tissues, with concentration of 31.8 ± 8.6 ng/g [88]. Toxicity studies with coral models, in larval and adult stages, were also performed, but the high variability and non-consistency of the results strongly suggested the need of more research to confirm the risk for corals [87,89,90]. Moreover, bioaccumulation could also affect the coral’s growth, inducing deformations and, in the last stage, coral’s mortality [56]. Indeed, BP-3 (**10**) and EHMC (**21**) were recently banned in sunscreens commercialised in Hawaii, due to these putative drastic consequences of coral bleaching [56,91]. Additionally, viral infections caused by UV filters may play a significant role in coral bleaching [92]. Despite the number of studies focused on organic UV filters, some inorganic UV filters could also have detrimental effects on corals. An example was the test performed with the coral species *Acropora* spp. exposed to ZnO (**35**) and modified particles of ZnO (**35**), which confirmed the bleaching caused by this specific UV filter. The modified particles (Eusolex^®^ T2000 and Optisol™) considerably diminished the bleaching of the coral, which proved the damaging effect caused by the non-modified ZnO (**35**) [59]. 

##### Other Marine Organisms

Bioaccumulation is the most referred effect in small marine organisms. Table 2 describes the negative effects of certain concentrations of UV filters, varying between ng/L and mg/L, in other marine organisms, such as algae (including microalgae and macroalgae), brine shrimp, crustaceans, dolphins, fish, and mussels. The studies reported negative effects in all the marine organisms due to certain concentrations of UV filters, namely organic UV filters, with exception for some corals [88] and mussels *Mylitus galloprovincialis* [53]. Additionally, many organic UV filters have been reported in collected marine samples, namely: homosalate (**8**) (algae and brine shrimp); benzophenone-1 (algae) and benzophenone-3 (**10**) (corals and algae); avobenzone (**11**) (algae, crustaceans, and brine shrimp); EHMC (**21**) (fish and mussels); octocrylene (**30**) (brine shrimp, crustaceans, and mussels); PABA (mussels) and camphor derivatives (fish). Growth inhibition was one of the negative effects that affects algae organisms, namely *Tetraselmis* sp. at concentrations of 1 mg/L for homosalate (**8**) [93] and green algae *Chlamydomonas reinhardtii* at 5 mg/L for benzophenone-1 and benzophenone-3 (**10**) [94]. Additionally, both benzophenones, at 5 mg/L, induced the decrease in photosynthetic pigments, and consequently create a deficit of nutrients essential for the growth and life of the marine algae *C. reinhardtii* [94]. As for marine algae *C. reinhardtii*, the effect of UV filters was also evaluated in *Artemia salina*, a species of brine shrimp. The results were determined after 48 h of exposure and revealed that homosalate (**8**), avobenzone (**11**), and octocrylene (**30**) induced mortality in 54%, 64%, and 88%, respectively, at concentrations of 2 mg/L, being octocrylene (**30**) considered the most toxic UV filter for this organism [93]. However, the environmental concentrations of these UV filters are around 500 times lower than those used in the studies. Avobenzone (**11**) (200 µg/L) and octocrylene (**30**) (200 µg/L) affected the crustacean *Daphia magna* by reducing its ability to detect or respond to light stimuli, thus highlighting some behavioural changes [95]. Neurotoxicity and malformations, decrease in heart rate and gene expression, as well as alteration in sexual differentiation were some of the consequences seen in zebrafish embryos *Danio renio* regarding to 3-(4-methylbenzylidene) camphor (**7**) at concentrations between 0.19 and 0.77 mg/L [96]. For fish *Oncorhynchus mykiss*, changes in metabolic pathways, increase in leukocytes and oxidative stress were detected, using common environmental concentrations (96.0–395.6 μg/kg) of EHMC (**21**) [60]. Despite this, no mortality and non-behavioral changes were observed [60]. EHMC (**21**), Padimate O (**24**), and octocrylene (**30**) were detected in Portuguese marine mussels, *M. galloprovincialis* and *M. edulis*, being octocrylene (**30**) detected in higher concentrations (2–7112 ng/g), in both organisms [14]. In addition, EHMC (**21**), PABA derivative (**24**), and octocrylene (**30**), were detected at 3992 ng/g dry weight (dw), 833 ng/g dw, and 1765 ng/g dw, respectively, in *M. galloprovincialis*, which evidenced the higher ability of these organisms to bioaccumulate these compounds [53]. Unexpectedly, octocrylene (**30**) was also detected at concentration of 782 ng/g in marine mammals, specifically *Pontoporia blainvillei* dolphins [63]. It is important to mention that the presence of UV filters in marine mammals raised the concern and supports the hypothesis of bioaccumulation and biomagnification of the UV filters through the food chain. Recently, Pawlowski et al. presented a tool (EcoSun Pass) that allows the evaluation of the eco-friendly profile of UV filters contained in photoprotective cosmetic formulations, especially sunscreen products [97], which could help avoiding the environmental hazard of future chemical substances, especially UV filters. 

## 3. Prospects

Several improvements in sunscreens were made in recent decades, particularly aiming to obtain new UV filters with increased photoprotective effectiveness, photostability, environmental and human safety, and improved sensory properties [98]. With this purpose, many sources have been explored by the scientific community, namely extracts and natural products isolated from both terrestrial and marine sources and new synthetic derivatives.

### 3.1. Nature as a Source of Potential Photoprotective Agents and UV Filters

Nature has been widely used for many decades, as the main source for the discovery of new bioactive compounds. Considering skin care applications, several botanical and marine organisms’ extracts with photoprotective and antioxidant effects have been reported. Additionally, some natural products isolated from these sources have proved to be promising bioactive compounds. 

Every day, plants are exposed to UVR, which increases their resistance to the noxious UV rays. As a result of this natural resistance, secondary metabolites with diversified scaffolds possessing UV photoprotective and antioxidant properties are produced, specially terpenoids, anthocyanins, flavonoids, carotenoids, and phenolic acids [21]. Algae, cyanobacteria, bacteria, and marine fungi are some of examples of marine organisms that produce secondary metabolites with photoprotective effects through UV filter and antioxidant activity. In Table 3, natural extracts and metabolites (36–56) of botanical and marine sources with photoprotective and antioxidant activities are presented. 

The by-products of wine, such as grape seeds [99,100], are considered a rich source of antioxidants, being most of those extracts constituted by polyphenolic compounds, namely flavonoids, including flavan-3-ol monomers, such as (+)-catechin (36) and ()-epicatechin (37), among others, as well as oligomers, as proanthocyanins [99]. Hubner et al. (2019) and Yarovaya et al. (2020) evaluated the grape seed extract as a potential sunscreen agent, focused on its photoprotective and photostability properties [99,100]. After biological tests, it was shown that the grape extracts restored the morphological characteristics of photo-damaged fibroblasts [99], and it was proven the synergistic antioxidant and photoprotection activities of phenolic compounds obtained from the extract of *Vitis vinifera L.* grapes [100], which demonstrated an amelioration in the solar protection factor (SPF) value of 81%. 

As for wine, olive oil by-products, such as olive leaves, also have a high content in polyphenols. Da Silva et al. (2019) studied the photoprotective and antioxidant properties of a commercial extract of *Olea europaea* with 20% of oleuropein (38) [101]. This extract displayed 1,1-diphenyl-2-picrylhydrazyl (DPPH) radical scavenging activity (IC_50_ = 11.75 μg/mL) and photoprotective activity exhibiting a SPF value of 22 and a maximum wavelength (λ_max_) of 376 nm [101]. It is noteworthy to mention that *V. vinifera* grape extracts [100] revealed a higher radical scavenging capacity than the commercial extract of olive leaves [101], probably due to the highest content in phenolic compounds. In addition, the SPF value evaluated for both extracts showed amelioration of photoprotective characteristics of grape extract [100] and the ability to prevent the skin damage in irradiated fibroblasts at 25 μg/mL [99]. 

Extracts of Brazilian *Lippia sericea* [102], Amazonian *Cecropia obtusa* [103], *Acacia catechu* heartwood [104], Brazilian bamboo species [105], and *Lasallia pustulata* [106] were also reported for their antioxidant and photoprotective activities. Considering *L. pustulata* extract, it was verified that gyrophoric acid (39) was the major component detected by HPLC, being proposed that this secondary metabolite should be the main responsible for the antioxidant and photoprotective activities [106]. The other extracts revealed to possess a complex mixture of compounds, namely a high content of phenols [102,105] and polyphenols [103] and high SPF values in formulations (SPF = 7–86) [102,103,104,105,106], being the extract of Brazilian bamboo species the most promising with a SPF value of 44, after irradiation [105]. Regarding leaves extract of *Cecropia obtusa*, it was demonstrated promising DPPH radical, superoxide radical, and singlet oxygen scavenging capacity (IC_50_ values of 1.63 µg/mL, 0.34 µg/mL, and 0.55 µg/mL, respectively), and the ability of maintaining the reactive oxygen species (ROS) species’ equilibrium in HaCaT cells [103]. Furthermore, a relation between the presence of a greater number of phenolic constituents in the extracts and the antioxidant activity was detected [102,103,104]. Alves et al. (2016) also proved the photoprotective effect of *C. obtusa* leaves extract at 20 µg/mL (SPF = 16) without cytotoxic effect in keratinocyte HaCaT cell line [103]. Additionally, the use of natural extracts of *A. catechu* heartwood [104] and wood powder [107] could be seen as a promising and eco-friendly approach in future sunscreens, since they improved the SPF value in formulation when used in 10% and 5%, respectively.

Rasheed et al. (2012) developed a herbal sunscreen with extracts of *Alpinia galanga*, *Curcuma longa,* and *Aloe vera,* and proved its efficacy against photo-induced damage [108]. Likewise, the ability to protect from UVR of coconut oil was also reported; however, more specific studies showed that the photoprotective ability was only towards UVC [109]. Bhattacharya and Sherje (2020) developed a hydro-gel formulation containing resveratrol (40) and green tea extract with improved SPF value (16.91), in contrast to the SPF obtained with formulations containing isolated samples of resveratrol (9.35) and green tea extract (14.59) [110]. It was also possible to enhance the efficiency of UV filters by increasing the SPF of the sunscreens, using vegetable oils [111,112], guava-fruit extract [113,114], rice bran and raspberry seeds [115], and red propolis extracts [116]. 

Marine organisms also produce bioactive compounds with high structural complexity, due to the stress conditions in which these secondary metabolites live. Among marine natural products, MAAs are well-known by their photoprotective characteristics (Table 3). There are more than 30 MAAs produced by algae [117] and cyanobacteria [118], but the most reported for their protective potential against UVR are palythine (41) [119], asterina-330 (42) [119,120], shinorine (43) [118,119], and porphyra-334 (45) [118]. Rangel et al. (2020) studied red macroalgae extracts from *Curdiea racovitzae* and *Iridaea cordata*, identifying, as major constituents, three MAAs, palythine (41), asterina-330 (42), and shinorine (43), which showed antioxidant, antiaging, and photoprotective effect in HaCaT keratinocytes, being demonstrated as non-cytotoxic and non-photounstable [119]. These MAAs were also found in an ethanolic extract of brown macroalgae *Sargassum cristafolium*, namely, palythine (41) [120]. 

Interestingly, scytonemin (44), a pigment and secondary metabolite produced by cyanobacteria *Stigonema* sp., *Scytonema* sp., and *Lyngbya* sp., was described as a potential broad-spectrum photoprotective agent due to its diverse maximum peak of absorbance (λ_max_ = 252, 278, 300, 386 nm) [121]. Recently, the promising anti-photoaging and anti-inflammatory activities of scytonemin (44) were described [122]. Other cyanobacteria, *Microcystis aeruginosa*, showed photoprotective characteristics as a UV absorber, with λ_max_ = 334 nm, due to MAAs shinorine (43) and porphyra-334 (45) [118]. 

Marine fungi *Penicillium echinulatum* was reported for producing photoprotective alkaloids (46–49), all the four metabolites being mainly absorbers of UVB and UVA radiation. Metabolites 48 and 49 were identified in major quantity in the collected extract, with critical wavelength (λ_c_) of 335 nm and 334 nm, respectively [123]. Among all the compounds, 49 was the most successful in inhibiting the generation of ROS through UVA-photoinduced damage in irradiated HaCaT keratinocyte cells, demonstrating its high potential as a future photoprotective compound. Five metabolites (50–54) produced by red algae *Bostrychia radicans*-associated fungus *Annulohypoxylon stygium* presented high photostability when irradiated, showing loss of absorbance (LoA) not higher than 5% [124]. However, when compared with compounds 46–49 [123], they possess phototoxicity, with photoirritation factor (PIF) values up to 5, with the exception of metabolites 50 and 51 [124].

Other marine organisms were investigated for their capacity to produce photoprotective metabolites, including the marine sea grass *Thalassia testudinum.* which demonstrated the ability to protect and repair the photoinduced UVB damage [125] due to the presence of a sulphated flavone glycoside thalassiolin B (55), and the platyfish *Xiphophorus*, capable of producing melanin (56), acting as an inhibitor of the formation of pyrimidine dimers, thus offering UV photoprotection [126].

Among all compounds from natural extracts indicated above, catechin (36), epicatechin (37), gyrophoric acid (39), and resveratrol (40) seem to be the most promising compounds obtained from botanical extracts, considering their good photostability, photoprotective, antioxidant potential, and non-cytotoxic profile at the concentrations mentioned. Interestingly, the presence of hydroxyl groups is a common structure feature for the most promising secondary metabolites with antioxidant activity. Considering marine-derived metabolites, MAAs, palythine (41), asterina-330 (42), shinorine (43), porphyra-334 (45), and scytonemin (44) have an excellent protective ability against UVR, being 44 an UVA/UVB absorber. Comparing both botanical and marine natural sources, marine-derived extracts and metabolites possess improved ability to protect against photo-induced damage. The antioxidant potential and easily introduction in cosmetic formulations are the main strengths of botanical extracts and metabolites. Despite all the advances on analytic techniques, it is not always possible to identify the active metabolite. Moreover, the isolation and purification of the botanical and marine natural products with photoprotective activity is a time-consuming process, being obtained in a low amount, which is a major drawback for obtaining compounds to be further explored for skin care applications. Therefore, some of these natural products were used as lead compounds to obtain synthetic derivatives with promising photoprotective effects.

**Table 3:** Natural extracts and metabolites of botanical and marine sources with photoprotective and antioxidant activity.

Organism and Species	Main Identified Secondary Metabolites	Activity	Values	References
Botanical Extracts and Metabolites				
Methanolic extract of grape seeds (from Village Farm and Winery; Nakhon Ratchasima, Thailand)	(+)-catechin (**36**) and (-)-epicatechin (**37**) (determined by HPLC) 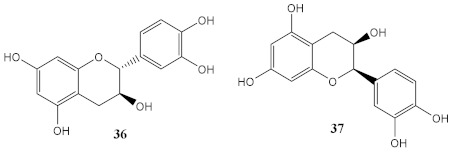	Photoprotective (% cell viability)	At 25 μg/mL 10 J/cm^2^ (110%) 20 J/cm^2^ (68%)	[99]
		Photodegradation	**36** = 35.1%; **37** = 31.3% Combination with UV filter: **36** (4.6%); **37** (7.0%)	
Hydroethanolic extract *of Vitis vinifera L*.	Flavonoids, phenolic compounds, procyanidins, among others (determined by HPLC)	Antioxidant (DPPH) at 1mg/mL	707.00 ± 0.03 µmol/g (pH = 5) 1098.00 ± 0.01 µmol/g (pH = 7)	[100]
		Photoprotection	SPF = 20–76 λ_c_ = 360–381 nm (pH = 5)	
Ethanolic commercial extract of olive leaves	20% of oleuropein (**38**) 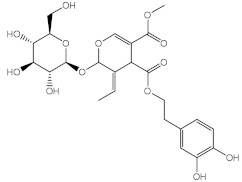	Antioxidant (DPPH)	38: IC_50_ = 11.75 ± 1.01 μg/mL Extract: IC_50_ = 13.8 ± 0.8 μg/mL	[101]
		Photoprotective	λ_max_ = 376 nm SPF = 22	
Ethanolic Extract of varied *Lippia* species (*L. brasiliensis*, *L. rotundifolia*, *L. rubella* and *L. sericea*)	Phenols and flavonoids	Antioxidant (DPPH)	IC_50_ = 0.604 mg/mL	[102]
		Photoprotective	SPF = 1.7–7.6 (formulation with 10% of the extract) λ_c_ = 375 nm	
Ethanolic extract of Amazonian *Cecropia obtusa* leaves	Polyphenols	Antioxidant	IC_50_ = 1.63 µg/mL (DPPH) IC_50_ = 0.34 µg/mL(O_2_^−^) IC_50_ = 0.55 µg/mL(^1^O_2_)	[103]
		Photoprotective	SPF = 16	
		Cytotoxicity (HaCaT keratinocyte cell line)	At 20 µg/mL: cell viability = 100%	
Ethanolic extract of *Acacia catechu* heartwood	-	Photoprotective	SPF = 24–30	[104]
Hydroalcoholic extract of five wild Brazilian bamboo species (*Chusqueaspp.*, *Aulonemia aristulata*, and *Merostachys pluriflora*)	Phenolic compounds	Antioxidant (DPPH)	IC_50_ = 137.55–260 μg/mL	[105]
		Photoprotective	SPF (before irradiation) = 34–86 SPF (after irradiation) = 14–44	
Dichloromethane/acetone (1:1) extract from *Lasallia pustulata*	Lichenic metabolites, being gyrophoric acid (**39**) identified by HPLC 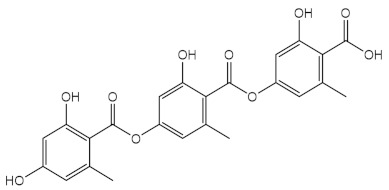	Antioxidant (DPPH)	25 % at 500 µg/mL	[106]
		Photoprotective	λ_max_ = 300 nm SPF = 5.03	
		Cytotoxicity (HaCaT keratinocytes cell line)	IC_50_ = 168 ± 33 µg/mL (before radiation) IC_50_ > 200 µg/mL (after radiation)	
Wood powder	-	Photoprotective	SPF = 11 (formulation) SPF = 37 (formulation + 5% of wood powder)	[107]
Ethanolic extracts of *Alpinia galanga*, *Curcuma longa* and *Aloe vera*	Flavonoids, phenols and terpenoids	Photoprotective	SPF = 18.2 (extract of *C. longa*) λ_max_ = 290 nm (*C. longa*) SPF = 15.1 (*A. galanga*) λ_max_ = 290 nm (*A. galanga*)	[108]
Coconut oil	High quantity of saturated fatty acids	Photoprotective	λ_max_ = 205 nm (coconut oil) λ_max_ = 320 nm (coconut oil + BP-3)	[109]
Resveratrol (**40**) and ethanolic extract of green tea	Resveratrol (**40**) 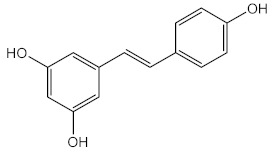	Antioxidant (DPPH)	IC_50_ = 38.67–85.44 % (resveratrol) IC_50_ = 37.41–77.50 % (green tea extract)	[110]
		Photoprotective	λ_max_ = 310 nm (**40**) λ_max_ = 270 nm (green tea) SPF = 9.35 (**40**) SPF = 14.59 (green tea extract) SPF = 16.91 (**40** and green tea extract)	
**Marine Organisms Extracts and Metabolites**				
Methanolic extract of red macroalgae *Curdiea racovitzae* and *Iridaea cordata*	MAAs, with major quantity of palythine (**41**), asterina-330 (**42**), and shinorine (**43**) 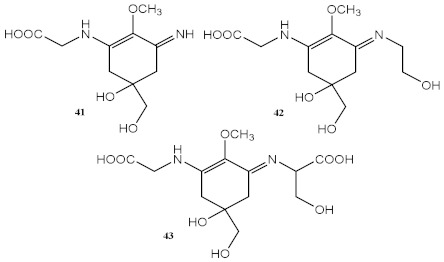	Antioxidant (DPPH)	IC_50_ = 970.00 μg/mL (*C. racovitzae*) IC_50_ = 2960.00 μg/mL (*I. cordata*)	[119]
		Photoprotective	λ_max_ = 320 nm (both) λ_c_ = 356 nm (*C. racovitzae*) λ_c_ = 347 nm (*I. cordata*)	
		Cytotoxicity (HaCaT keratinocytes cell line)	At 1 mg/mL % cell viability = 89 (*C. racovitzae*) % cell viability = 73 (*I. cordata*)	
Ethanolic extract of brown macroalgae *Sargassum cristafolium*	Palythine (**41**)	Photoprotective	λ_c_ = 370 nm	[120]
Methanolic extract red alga *Corallina pilulifera*	-	Antioxidant (DPPH)	At 200 mg/mL: 80% scaveging activity	[127]
Metabolite from extracts of cyanobacteria *Stigonema* sp., *Scytonema* sp. and *Lyngbya* sp.	Scytonemin (**44**) 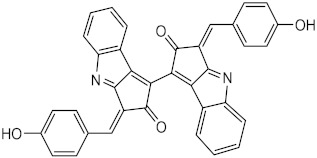	Photoprotective	λ_max_ = 252, 278, 300, 386 nm	[121]
Metabolites from aqueous methanolic extract of cyanobacteria *Microcystis aeruginosa*	MAAs shinorine (**43**) and porphyra-334 (**45**) 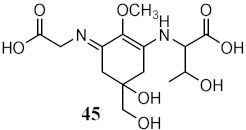	Photoprotective	λ_max_ = 334 nm	[118]
Metabolites from ethyl acetate extract of marine fungi *Penicillium echinulatum*	Quinolinic Alkaloids 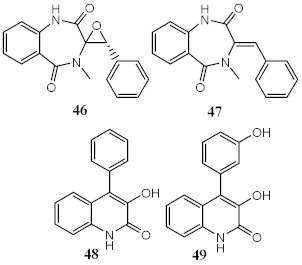	Photoprotective	λ_max_ = 287 (**48**) λ_c_ = 335 nm (**48**) λ_max_ = 330 (**49**) λ_c_ = 334 nm (**49**)	[123]
		Phototoxicity (HaCaT keratinocytes cells)	Reduction of ROS (43%) at 200 µg/mL (**49**)	
Metabolites from dichloromethane/methanol (2:1) extract of algae *Bostrychia radicans* -associated fungi *Annulohypoxylon stygium*	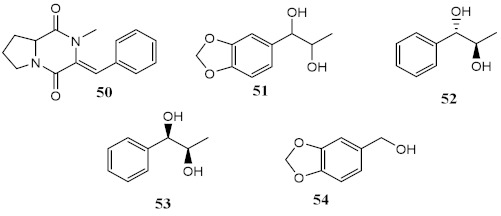	Phototoxicity (3T3 murine fibroblasts)	PIF = 1.00 (**50** and **51**) PIF = 5.2 (**54**)	[124]
Metabolite from ethanolic extract of plant *Thalassia testudinum*	Thalassiolin B (**55**) 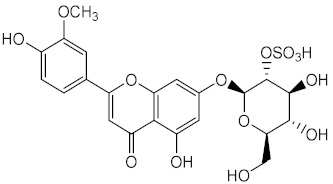	Antioxidant (DPPH)	IC_50_ = 100 μg/mL	[125]
		Repair of Acute UVB-Damaged Skin	Skin damage suppression (with **55** at 240 μg/cm^2^) = 90%	
Platyfish *Xiphophorus* metabolite	Melanin (**56**) 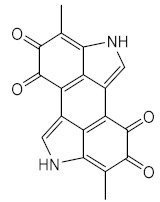	Photo-repair of the skin	Stimulate the production of melanin, which reduced the formation of pyrimidine dimers.	[126]

Abbreviations: DPPH—2,2-diphenyl-1-picrylhydrazyl; SPF–solar factor protection; λ_c_—critical wavelength; λ_max_—maximum wavelength; IC_50_—concentration that reduces a response to 50% of its maximum; HPLC—high-performance liquid chromatography; PIF—photoirritation factor.

### 3.2. Synthetic Derivatives with Photoprotective and UV Filter Activity

#### 3.2.1. Inorganic UV Filters

Cerium oxide (CeO_2_) was suggested as one possible UV filter. In fact, it is commercialised in some photoprotective formulations but with silica coating, due to its high photocatalytic activity, responsible for oxidation and degradation of other formulations’ components [128]. The coating with amorphous silica also decreases its photoprotective UV-shielding potential [128]. However, this gap could be ameliorated if CeO_2_ was doped with Ca^2+^ and Zn^2+^ ions, which could reduce its photocatalytic activity and particle size, without interfering with its photoprotective potential [128]. Cerium phosphate (CePO_4_) was reported 10 years later by Seixas and Serra (2014) [129]. Similarly to CeO_2_, CePO_4_ was described for possessing high photocatalytic activity, low amount of white residue when applied on the skin, and increased stability [129]. Some parameters regarding physical and chemical stability of CePO_4_ were evaluated, using TiO_2_ (**34**) and ZnO (**35**) as controls, as well as its behavioural and rheological properties, both alone and in formulation. The results revealed low interaction in formulation when combined with organic UV filters. Therefore, CePO_4_ is a potential future novel, stable, and efficient inorganic UV filter [129].

#### 3.2.2. Organic UV Filters

**Figure 5:**
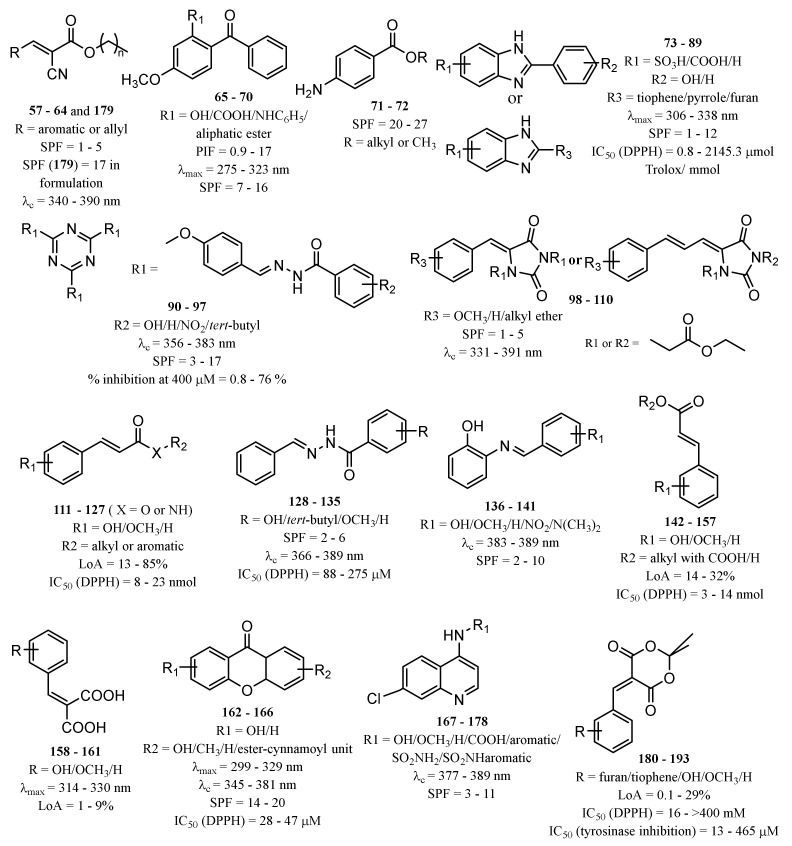
Chemical skeleton and values of biological activity assessed for the synthetic derivatives reported in the literature.

Inspired by commercialised UV filters, as well as in natural products with photoprotective properties, several compounds have been synthesised and reported as potential UV filters, with antioxidant, anti-inflammatory, and anti-photoaging activities. Herein, a reference of the novel synthetic derivatives developed with the aim of obtaining UV filtering compounds with extra pharmacological properties are presented. Table S2 summarises the structures and the biological data reported for synthetic derivatives with photoprotective and antioxidant activities, as potential UV filters, and Figure 5 presents the chemical skeleton, and the range of values obtained for the biological activity assessed.

##### New Synthetic Derivatives Inspired by Commercialised UV Filters

One of the strategies followed by research groups to obtain new organic UV filters with improved photoprotective activity is through molecular modifications of actually marketed UV filters. Eight octocrylene (**30**)-related compounds (**57**–**64**) were prepared and evaluated for their photoprotective effect by Polonini et al. (2014) [130]. Among these, **60**, **61**, and **63** displayed the best UVB protection effect, while compounds **61**–**63** presented the best results concerning protection against UVA. The most promising derivative was **63**, which behaved as a broad band UVA/UVB filter [130]. 

Using benzophenone derivatives as lead compounds, benzophenones **65**–**68** were prepared and tested for their UV filtering properties [131].Compounds **65** and **66** were considered as the most promising, showing photoprotective activity and non-phototoxic results, confirmed by PIF values as less than 1.3. In addition to these benzophenones, the structure-related benzophenone **69** and lactone **70** displayed UV filter properties, having lactone **70,** a more potent photoprotective effect (SPF = 16), but only ability to absorb UVB radiation, contrarily to benzophenone **69**, which demonstrated the ability to absorb UVA radiation [132]. 

Later, new PABA derivatives, PABA methyl ester (**71**) and PABA methyl stearate (**72**), were prepared and evaluated for their photoprotective potential, revealing SPF values of 20.60 and 26.17, respectively [133]. It is noteworthy to mention that the high molecular weight of **72** should avoid its penetration through the skin, making this compound a potential UV filter with a safer profile.

Inspired by benzimidazole and benzotriazole approved UV filters, several new heterocyclic compounds were prepared. Benzimidazole derivatives **73**–**86** were reported for their photoprotective activity, and compound **83** also demonstrated antioxidant activity and higher photostability (98.4%) when compared with the control phenylbenzimidazole sulfonic acid (PBSA) (**14**) (96.7%) [134]. Additionally, new 5-membered ring-benzimidazole derivatives (**87**–**89**) were also prepared, being compounds with pyrrole (**87**), furan (**88**), and thiophene (**89**) moieties which were the most promising regarding their antioxidant, photostability, and photoprotective activities [135]. Among these, the most photostable was the thiophene derivative **89**, followed by pyrrole derivative **87**, and the furan derivative **88** [135].

Using triazine UV filters as models, new 1,3,5-triazine derivatives (**90**–**97**) were synthesised and evaluated for their photoprotective properties [25]. Among 1,3,5-triazine derivatives **90**–**97**, **97** displayed the most promising antioxidant activity and revealed the highest SPF and UVA protection factor [25]. 

Inspired in 3-benzylidenecamphor (**7**), Popiół et al. (2019) planned a small library of potential UV filters (**98110)** by replacing the camphor moiety by 5-arylideneimidazolidine-2,4-dione (hydantoin) while maintaining the benzylidene portion. Although the synthesised compounds revealed moderate SPF values, they demonstrated the ability to absorb both UVA and UVB radiation (λ_c_ between 339 and 391 nm) [136]. Compounds **104** and **109** were considered the less toxic against HaCaT keratinocytes and human fibroblasts cell lines, and compound **99** revealed the best UVB photoprotective properties within the tested series, with a SPF = 4.7. Some structure–activity relationships (SAR) considerations could be drawn for these derivatives. For instance, methoxy substituents at the aromatic ring are associated with photoprotection against UVA radiation; in contrast, the absence of methoxy groups in the aromatic ring is associated with interesting UVB filter properties [136]. In addition, the presence of alkoxy groups at positions 4- (compounds **99**, **104**, and **109**) and 3,4- (compounds **102** and **107**) is associated with the highest values of critical wavelength, contrarily to what is observed with non-substituted benzene rings [136].

Sinapic acid analogues of EHMC (**21**) with ester (**111**–**124)**, amide (**125**–**126**), and ketone (**127**) groups revealed promising UV filter activity. Interestingly, **111** and **114**–**127** showed multifunctional properties, combining antioxidant and photoprotective effects. Among all compounds, the derivatives **125** and **127** showed the best antioxidant activities with IC_50_ values lower than 8.9 ± 0.3 nmol [137]. Additionally, sinapic acid analogue **111** and its methylated derivative **112**, aliphatic sinapate derivatives **116** and **118,** and amide derivative **125** presented higher photostability than EHMC (**21**) [137]. 

Molecular hybridisation avobenzone (**11**), EHMC (**21**), and *trans*-resveratrol (**40**) resulted in the identification of a novel series of hybrids (**128**–**135**) with UV filter effect [138]. All compounds revealed photoprotective activity with SPF values varied between 2 and 5, and an ability to absorb the UVA region of the electromagnetic spectrum, confirmed by their λ_max_ values in the range of 369 nm and 389 nm [138]. Additionally, three hybrids of the total synthetised compounds **128**–**135** possess antioxidant potential, with an IC_50_ between 88 µM and 275 µM (compounds **128**, **134**, and **135**) [138]. Amongst all, compounds **128** and **131**–**135** possess characteristics of broad-spectrum molecules, having **128**, **134**, and **135** an interesting DPPH radical scavenging activity.

##### Nature-Inspired Synthetised Compounds

Naturally occurring stilbenes, *p*-hydroxycinnamic acids, and xanthones have been used as inspiration to obtain new potential UV filters. Inspired in the photoprotective activity of *trans*-resveratrol (**40**), compounds **126**–**141** were prepared and tested for their UV filter effect. All compounds revealed promising UV filter properties, with SPF values between 2 and 20 [139].

Sinapoyl-*L*-malate (**142**) is a sinapoyl ester widely described for its UV protection in plants [140]. Taking this into account, the UV filter activity of sinapoyl-*L*-malate **142** and its analogues **143**–**157** were explored by Peyrot et al. (2020) [24]. All compounds presented good water solubility, as a result of the presence of a free carboxylic acid in their structure which could facilitate the incorporation into sunscreen’s formulation. Among all the compounds, **142**–**157**, **142**, **144**, **147**, and **150**–**157** showed promising photoprotective activity with LoA < avobenzone (**11**), and antioxidant activity, being **151** and **155**–**157** the most promising. Moreover, **142**, **151,** and **154**–**157** revealed photostability with LoA values less than 20% [24].

Based in natural-inspired *p*-hydroxycinnamic acids, *p*-hydroxycinnamic diacids were prepared (**158**–**161**), being sinapic diacid (**160**) and caffeic diacid (**161**) the derivatives that displayed the best photoprotective characteristics [141].

Xanthone derivatives were studied in order to disclose their profiles as future UV filtering molecules. Resende et al. (2020) reported three hydroxylated xanthone derivatives (**162**–**164**) with promising antioxidant activity and UV filtering characteristics [142]. Compounds **162**–**164** proved to absorb in the UVB range (280–320 nm). Additionally, xanthone **162** showed a dual ability to protect the skin against UV damage, through DPPH scavenging action and UV-filter capacity, without phototoxicity in the HaCaT keratinocyte cell line [142]. Popiół et al. (2021) also reported novel potential and innovative UV filtering compounds, combining the xanthone scaffold with €-cinnamoyl moiety [26]. Active xanthone-cinnamoyl hybrid compounds **165** and **166** were synthetised and evaluated for their photoprotective, antioxidant, and mutagenic activities [26]. Compound **166** was revealed to be the most promising, displaying λ_c_ of 381 nm, confirming the ability to absorb both UVA and UVB radiations and with a SPF of 19.69 [26]. Comparing these two groups of xanthones, the combination of the cinnamoyl and xanthonic moieties allows the correct electronic delocalisation, which improves the UV absorber properties and, because of that, compounds reported by Popiół et al. [26] possess action against UVA and UVB radiation, in contrast to compounds with a simple xanthone scaffold reported by Resende et al. [142]. 

##### Other New Synthetic Derivatives 

Other potential synthetic UV filters with different scaffolds have been described, namely those with heterocyclic rings, such as the new UV absorbers **167**–**178** based on quinoline derivatives with SPF and λ_c_ values between 2 and 11 and 376 and 388 nm, respectively, being the quinoline derivative **176** considered as the most promising compound with the highest SPF value [143]. (*E*,*Z*)-2-ethylhexyl-2-cyano-3-(furan-2-yl)acrylate (**179**) has also been described for its good capacity to absorb UVA radiation (λ_max_ = 339 nm) and good solubility in oils for the formulation [144]. 

Recently, Peyrot et al. (2020) developed a small library of compounds from Meldrum’s acid and *p*-hydroxycinnamic acids (**180**–**183**), furans (**184**–**190**), and pyrroles (**191**–**193**), displaying interesting UV filter properties and photostability. Moreover, *p*-hydroxycinnamic acid-based Meldrum’s derivatives (**180** and **183**) possess antioxidant and anti-tyrosinase properties, photoprotective characteristics, namely against UVA radiation and blue light, and photostability (with LoA < avobenzone (**11**)) [145] reinforcing their potential as multifunctional agents for cosmetic application [24]. Interestingly, endocrine disruption assays were performed for compounds **182**, **184**, **187**, and **191**, that revealed non-interaction with receptors, showing the absence of agonistic (% receptor activity < 30%) and antagonistic (% receptor activity > 70%) effects [145]. 

## 4. Conclusions

Ultraviolet filters are incorporated in sunscreens aiming to protect the skin from the noxious effects of UV rays. Despite the strict regulation framework, new scientific evidences have raised concern about their toxic effects in humans and marine ecosystems. Neurotoxicity, endocrine disruption, malformations, decreased photosynthetic pigments, coral bleaching, and mortality, among others, are some of the confirmed negative effects that some UV filters, namely benzophenone-3 (**10**), avobenzone (**11**), EHMC (**21**) octocrylene (**30**), can have in marine organisms. The decomposition of the UV filters detected in aquatic ambient was already reported, leading to the formation of toxic by-products with putative negative effects for human beings and accumulation in marine organisms. Beyond these environmental problems, UV filters can also have direct negative effects on humans, especially when photodegradation/photoisomerisation occurs. Avobenzone (**11**) is the UV filter most studied regarding to its photoinstability and negative effects, hence being one of the most toxic UV filters when exposed to UV radiation. The presence of certain chemical groups, such as aromatic ketones, unsaturated systems, and camphor structure, are some of the chemical moieties susceptible of inducing allergic and sensitisation skin reactions.

Considering the mentioned pitfalls, the scientific community has been focused on creating new UV filters. The presence of labile groups suitable for hydrolysis degradation could be an approach, known in pharmaceutical sciences as “soft drugs”, aiming towards the degradation of the parent compound into inactive metabolites avoiding the oxidative pathway, and contributing to a decrease in bioaccumulation and toxicity. 

The existence of privileged structures in nature, produced by plants and marine organisms, is vastly known. Natural products could be directly used, after their extraction, or could inspire the creative mind of the scientists to obtain synthetic derivatives with improved efficacy and safer profile. Botanical extracts and metabolites, namely catechin (**36**), epicatechin (**37**), gyrophoric acid (**39**), and resveratrol (**40**), are some of the plant-derived metabolites that could be highlighted for their photoprotective ability, but especially for their antioxidant potential due to the presence of hydroxyl groups in their structure. In addition to botanical extracts, marine secondary metabolites also exhibit photoprotection properties, namely MAAs, which are able to absorb both UVA and UVB radiation. Some derivatives inspired by marketed UV filters were also developed to overcome some of their drawbacks. From all the synthetic series presented, the camphor (**7**)- and avobenzone (**11**)-inspired derivatives, which are safer and photostable, and octocrylene (**30**)-inspired derivatives with improved photoprotective performance can be highlighted. Considering synthetic derivatives, benzimidazole, *p*-hydrocinnamic acids, quinolines, and xanthones are the base-chemical scaffolds that showed dual photoprotective–antioxidant activities. Molecular modifications are an interesting and simpler strategy to ameliorate some of characteristics of the actual commercialised UV filters. Aromatic systems, such as benzimidazole (**75** and **87**–**89**), triazine (**93**), xanthone (**162** and **166**), and quinoline (**176**) derivatives, are crucial for the good absorption in UV region. Additionally, the presence of other chromophores, such as the linkers that join several of these structures, with ester, amide, hydrazine groups, and double and/or triple bonds, ensure an extra effect in the final structure of the compounds, contributing to a higher ability to absorb in UVA region. Substituent groups in the aromatic scaffold, such as OH, OCH_3_, NH_2_, CN, and SO_3_H, among others, have been revealed to be favourable regarding the photoprotective activity, due to the presence of electronegative atoms that also allow the electronic delocalisation after irradiation. These highlighted compounds are some of the most promising in the series of the almost 150 synthetised molecules herein presented. With the aim of obtaining multifunctional compounds, the presence of at least one hydroxyl group is essential for antioxidant activity, the catechol group and 3,5-dimethoxy-4-hydroxyl pattern being the most favourable substitution moieties in the aromatic rings, which each revealed interesting antioxidant potential, namely as scavenger of the DPPH radical. Particularly *p*-hydroxycinnamic acid moiety and *p*-hydroxycinnamic diacids, compounds **151**, **155**–**157** and **160,** and **161**, **180**, and **183** can be highlighted as molecules with antioxidant and photoprotective activities, presenting some of them an extra ability as solubilising agents, when added to a sunscreen formulation (**151** and **155**–**157**).

To conclude, the development of innovative, safe, effective, and non-toxic UV filters is an ongoing need and a hot research topic. Taken together, all the strategies presented in this work represent a helpful insight for the creation of the next generation UV filters with attractive and essential features.

## Figures and Tables

**Table 2:** Summary of the negative impact of UV Filters in marine organisms.

Marine Organism	Species	UV Filter	Concentration	Negative Effects	Reference
Corals	*Pocillopora damicornis*, *Seriatopora caliendrum*, *Stylophora pistillata*, *Acropora* spp.	Benzophenone-3 (**10**)	31.8 ng/g	Not described	[88]
	*Acropora* spp.	Zinc Oxide (**35**)	-	Coral bleaching	[59]
Algae	*Tetraselmis* sp.	Homosalate (**8**)	1 mg/L	Growth inhibition	[93]
			-	Changes in cell morphology	
		Benzophenone-3 (**10**)			
		Avobenzone (**11**)			
	*Chlamydomonas reinhardtii*	Benzophenone-1	5 mg/L	Growth inhibition Decrease of photosynthetic pigments	[94]
		Benzophenone-3 (**10**)			
Brine Shrimp	*Artemia salina*	Homosalate (*8*)	2 mg/L	Induce mortality in 54%	[93]
		Avobenzone (**11**)		Induce mortality in 64%	
		Octocrylene (**30**)		Induce mortality in 88%	
Crustaceans	*Daphnia magna*	Avobenzone (**11**)	200 μg/L	Induce metabolic disruption Reduce ability to detect light stimuli Behavioural changes	[95]
		Octocrylene (**30**)	200 μg/L		
Dolphins	*Pontoporia blainvillei*	Octocrylene (**30**)	782 ng/g	Bioaccumulation and biomagnification	[63]
Fish	*Danio rerio*	3-(4-methylbenzylidene) camphor (**7**)	0.19–0.77 mg/L	Induce malformations Decrease heart rate Affecting sexual differentiation Induce neurotoxicity	[96]
	*Oncorhynchus mykiss*	Ethylhexyl methoxycinnamate (**21**)	96.0–395.6 μg/kg	Changes in metabolic pathway Increasing of leukocytes Oxidative stress	[60]
Mussels	*Mytilus galloprovincialis*	Benzophenone-3 (**10**)	100 µg/L	Affect the metabolic activity	[52]
			1000 µg/L	Induce cellular damage	
		Ethylhexyl methoxycinnamate (**21**)	3992 ng/g	Not described	[53]
		Padimate O (**24**)	833 ng/g		
		Octocrylene (**30**)	1765 ng/g		
		Ethylhexyl methoxycinnamate (**21**)	3–256 ng/g	Bioaccumulation	[14]
		Octocrylene (**30**)	2–7112 ng/g		
	*Mytilus edulis*	Ethylhexyl methoxycinnamate (**21**)	3–256 ng/g	Bioaccumulation	[14]
		Octocrylene (**30**)	2–7112 ng/g		

## Data Availability

Not applicable.

## Supplementary Materials

The following are available online at https://www.mdpi.com/article/10.3390/ph15030263/s1, Table S1: List of UV filters approved for use in cosmetics products in EU in 2009 (actualised version in October 2021), Table S2: Synthetic derivatives with photoprotective and antioxidant activities, as potential UV filters.
