# Supplementary material for: UV Filters: Challenges and Prospects
Source: Pharmaceuticals (Basel). 2022 Feb 22;15(3):263. doi: 10.3390/ph15030263 (PMC8955451; doi:10.3390/ph15030263)
Supplement: Supplementary file 1 [file pharmaceuticals-15-00263-s001.zip › pharmaceuticals-1551555-supplementary.pdf]

*pharmaceuticals*

*Review*

# UV Filters: Challenges and Prospects

**Ana Jesus [1,2], Emília Sousa [3,4], Maria T. Cruz [5,6], Honorina Cidade [3,4,*], José M. Sousa Lobo[1,2] and Isabel F. Almeida [1,2*]**

[1] UCIBIO – Applied Molecular Biosciences Unit, MedTech, Laboratory of Pharmaceutical Technology, Department of Drug Sciences, Faculty of Pharmacy, University of Porto, 4050-313 Porto, Portugal. anaaimjesus@gmail.com (A.J.).
[2] Associate Laboratory i4HB - Institute for Health and Bioeconomy, Faculty of Pharmacy, University of Porto, 4050-313 Porto, Portugal
[3] Laboratory of Organic and Pharmaceutical Chemistry, Department of Chemical Sciences, Faculty of Pharmacy, University of Porto, 4050-313 Porto, Portugal; esousa@ff.up.pt (E.S.).
[4] CIIMAR–Interdisciplinary Centre of Marine and Environmental Research, Avenida General Norton de Matos, S/N, 4450-208 Matosinhos, Portugal
[5] Faculty of Pharmacy, University of Coimbra, 3004-531 Coimbra, Portugal; trosete@ff.uc.pt (M.T.R.).
[6] Center for Neuroscience and Cell Biology, 3004-504 Coimbra, Portugal.
* Correspondence: hcidade@ff.up.pt (H.C.); ifalmeida@ff.up.pt (I.F.A.); Tel.: +351-220-428 (I.F.A.)

**Table of contents:**

**Table S1.** List of UV filters approved for use in cosmetics products in EU in 2009 (actualized version in October 2021).

***Table S1.*** *List of UV filters approved for use in cosmetics products in EU in 2009 (actualized version in October 2021).*

| Number | Chemical structure | Chemical name[a] | INCI name and Abbreviation [b] | Maximum concentration allowed[c] | Characteristics[a,c] | Molecular weight (g/mol) [a] |
|---|---|---|---|---|---|---|
| | | **Organic UV Filters** | | | | |
| | | **Camphor derivatives** | | | | |
| **1** | 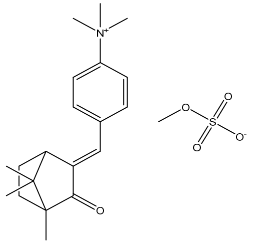 | *N,N,N*-trimethyl-4-[(*E*)-(4,7,7-trimethyl-3-oxobicyclo[2.2.1]hept-2-ylidene)methyl]anilinium methyl sulfate | Camphor benzalkonium methosulfate (CBM) | 6% | Organic UVB absorber | 409.54 |
| **2** | 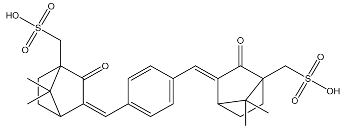 | [(3*E*)-3-[[4-[(*Z*)-[7,7-dimethyl-3-oxo-4-(sulfomethyl)-2-bicyclo[2.2.1]heptanylidene]methyl]phenyl]methylidene]-7,7-dimethyl-2-oxo-1-bicyclo[2.2.1]heptanyl]methanesulfonic acid | Terephthalydene dicamphor sulphonic acid/ Ecamsule (TDSA) and its sodium and amine salts | 10% | Organic UVA absorber | 562.69 |
| **3** | 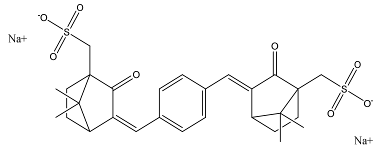 | disodium;[(3*E*)-3-[[4-[(*Z*)-[7,7-dimethyl-3-oxo-4-(sulfonatomethyl)-2-bicyclo[2.2.1]heptanylidene]methyl]phenyl]methylidene]-7,7-dimethyl-2-oxo-1-bicyclo[2.2.1]heptanyl]methanesulfonate | | | | 606.7 |
| **4** | 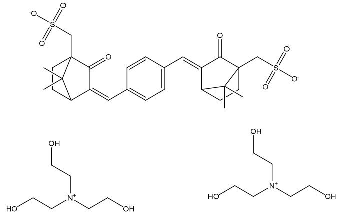 | 2-[bis(2-hydroxyethyl)amino]ethanol;[3-[[4-[[7,7-dimethyl-3-oxo-4-(sulfomethyl)-2-bicyclo[2.2.1]heptanylidene]methyl]phenyl]methylidene]-7,7-dimethyl-2-oxo-1-bicyclo[2.2.1]heptanyl]methanesulfonic acid | | | | 861.1 |
| **5** | 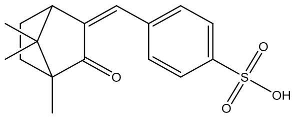 | 4-[(4,7,7-trimethyl-3-oxo-2-bicyclo[2.2.1]heptanylidene)methyl]benzenesulfonic acid | Benzylidene camphor sulfonic acid (BCSA) | 6% | Organic UVB absorber | 320.40 |

| | | | | | | |
|---|---|---|---|---|---|---|
| **6** | 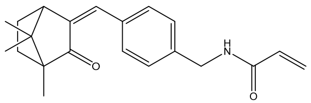 | Polymer of *N*-{(2 and 4)-[(2-oxoborn-3-ylidene)methyl]benzyl}acrylamide 2-Propenamide,*N*-[[4-[(4,7,7-trimethyl-3-oxobicyclo[2.2.1]hept-2-ylidene)methyl]phenyl]methyl]-, homopolymer | Polyacrylamidomethyl benzylidene camphor (PBC) | 6% | Organic UVB absorber | 323.44 |
| **7** | 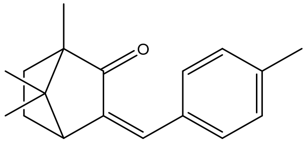 | (3*E*)-1,7,7-trimethyl-3-[(4-methylphenyl)methylidene]bicyclo[2.2.1]heptan-2-one | 4-Methylbenzylidene Camphor/ Enzacamene (4-MBC) | 4% | Organic UVB and UVA absorbers | 254.37 |
| **Salycilate derivatives** | | | | | | |
| **8** | 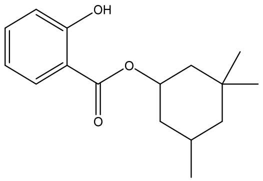 | (3,3,5-trimethylcyclohexyl) 2-hydroxybenzoate | Homosalate (HMS) | 10% | Organic UVB and UVA absorber | 262.35 |
| **9** | 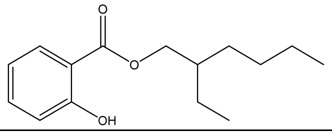 | 2-ethylhexyl 2-hydroxybenzoate | Ethylhexyl salicylate/ octisalate (EHS) | 5% | Organic UVB and UVA absorber | 250.34 |
| **Benzophenone and dibenzoylmethane derivatives** | | | | | | |
| **10** | 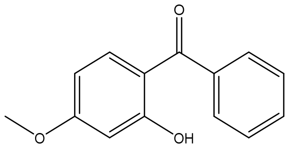 | (2-hydroxy-4-methoxyphenyl)(phenyl) methanone/ oxybenzone | Benzophenone-3 (BP-3) | 6% | Organic UVB and UVA absorber Benzophenone derivative | 228.25 |
| **11** | 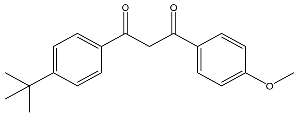 | 1-(4-*tert*-butylphenyl)-3-(4-methoxyphenyl)propane-1,3-dione | Butyl methoxydibenzoylmethane/ avobenzone (BMDBM) | 5% | Organic UVB and UVA absorber | 310.4 |

| | | | | | | |
|---|---|---|---|---|---|---|
| | | | | | Dibenzoylmethane derivative | |
| **12** | 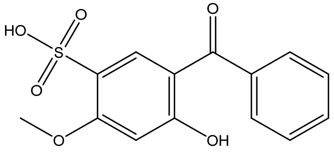 | 5-benzoyl-4-hydroxy-2-methoxybenzene-1-sulfonic acid | Benzophenone-4/ sulisobenzone (BP-4) | 5% | Organic UVB and UVA absorber Benzophenone derivatives | 308.30 |
| **13** | 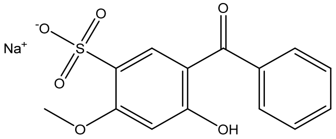 | sodium;5-benzoyl-4-hydroxy-2-methoxybenzenesulfonate | Benzophenone-5 (BP-5) | | | 330.29 |
| **Benzimidazole and Benzotriazole derivatives** | | | | | | |
| **14** | 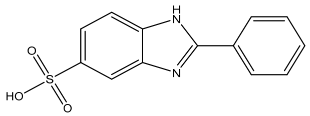 | 2-phenyl-1*H*-1,3-benzodiazole-5-sulfonic acid | | | | 274.29 |
| **15** | 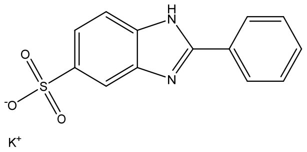 | Potassium 2-phenyl-1*H*-1,3-benzodiazole-5-sulfonate | Phenyllbenzimidazole sulfonic acid/ Ensulizole (PBSA) | 8% | Organic UVB and UVA absorber Benzimidazole derivative | 312.38 |
| **16** | 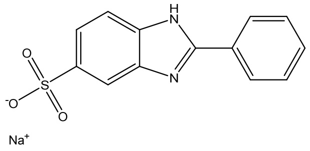 | Sodium 2-phenyl-1*H*-1,3-benzodiazole-5-sulfonate | | | | 296.28 |

| | | | | | | |
|---|---|---|---|---|---|---|
| **17** | 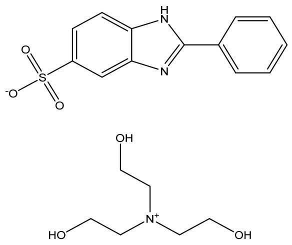 | Triethanolamine 2-phenyl-1*H*-1,3-benzodiazole-5-sulfonate | | | | 423.48 |
| **18** | 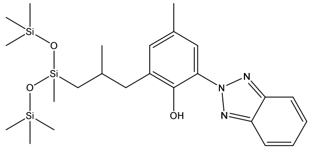 | 2-(benzotriazol-2-yl)-4-methyl-6-[2-methyl-3-[methyl-bis(trimethylsilyloxy)silyl]propyl]phenol | Drometrizole Trisiloxane (DRT) | 15% | Organic UVB and UVA absorbers Benzotriazole derivative | 501.85 |
| **19** | 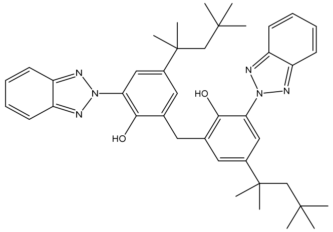 | 2-(benzotriazol-2-yl)-6-[[3-(benzotriazol-2-yl)-2-hydroxy-5-(2,4,4-trimethylpentan-2-yl)phenyl]methyl]-4-(2,4,4-trimethylpentan-2-yl)phenol | Bisoctrizole/ Bisoctrizole (nano) (MBBT) | 10% | Organic UVB and UVA absorber Benzotriazole derivative | 658.89 |
| **20** | 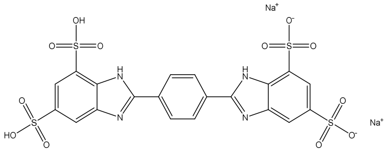 | disodium;6-sulfo-2-[4-(6-sulfo-4-sulfonato-1*H*-benzimidazol-2-yl)phenyl]-1*H*-benzimidazole-4-sulfonate | Bisdisulizole disodium (DPDT) | 10% | Organic UVA absorber Benzimidazole derivative | 674.55 |
| | | **Cinnamate deriatives** | | | | |
| **21** | 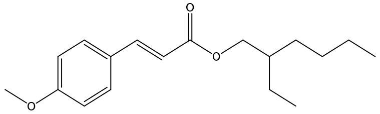 | 2-ethylhexyl-4-methoxycinnamate | Ethylhexyl methoxycinnamate/ octinoxate (EHMC) | 10% | Organic UVB and UVA absorber | 290.40 |

| | | | | | | |
|---|---|---|---|---|---|---|
| **22** | 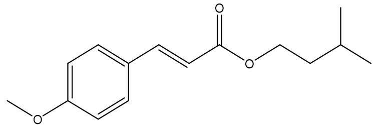 | 3-methylbutyl (*E*)-3-(4-methoxyphenyl)prop-2-enoate | Isoamyl p-methoxycinnamate/ amiloxate (IMC) | 10% | Organic UVB and UVA absorber | 248.32 |
| | | **PABA derivatives** | | | | |
| **23** | 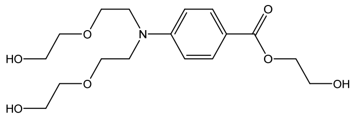 | 2-hydroxyethyl 4-(bis(2-(2-hydroxyethoxy)ethyl)amino)benzoate | PEG-25 PABA | 10% | Organic UVB absorber | 357.40 |
| **24** | 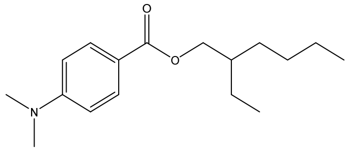 | 2-ethylhexyl 4-(dimethylamino)benzoate | Padimate O (EHDB) | 8% | Organic UVB absorber | 277.41 |
| | | **Triazine and Triazone Derivatives** | | | | |
| **25** | 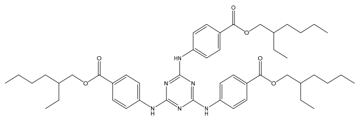 | 2-ethylhexyl 4-[[4,6-bis[4-(2-ethylhexoxycarbonyl)anilino]-1,3,5-triazin-2-yl]amino]benzoate | Octyl triazone/ Ethylhexyl triazone (OT/ EHT) | 5% | Organic UVB and UVA absorber Triazone derivative | 823.09 |
| **26** | 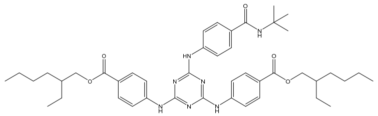 | 2-ethylhexyl 4-[(4-{[4-(tert-butylcarbamoyl)phenyl]amino}-6-[(4-{[(2-ethylhexyl)oxy]carbonyl}phenyl)amino]-1,3,5-triazin-2-yl)amino]benzoate | Diethylhexyl Butamido Triazone/ Iscotrizinol (DBT) | 10% | Organic UVB and UVA absorbers Triazone derivative | 765.98 |
| **27** | 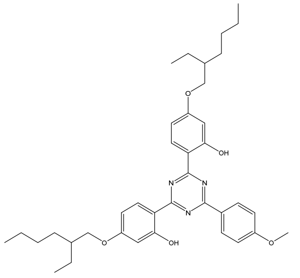 | 5-(2-ethylhexoxy)-2-[4-[4-(2-ethylhexoxy)-2-hydroxyphenyl]-6-(4-methoxyphenyl)-1,3,5-triazin-2-yl]phenol | Bemotrizinol (BEMT) | 10% | Organic UVB and UVA absorber Triazine derivative | 627.83 |

| | | | | | | |
|---|---|---|---|---|---|---|
| **28** | 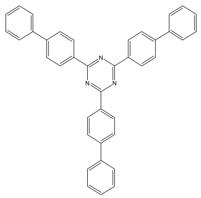 | 2,4,6-tris(4-phenylphenyl)-1,3,5-triazine | Tris-biphenyl triazine and Tris-biphenyl triazine (nano) (TBT/ TBPT) | 10% | Organic<br>UVB and UVA absorber<br>Triazine derivative | 537.67 |
| **29** | 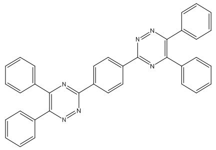 | 3,3′-(1,4-phenylene)bis(5,6-diphenyl-1,2,4-triazine) | Phenylene Bis-Diphenyl triazine | 5% | Organic<br>Triazine derivative<br>UVA and UVB absorber<br>Triazine derivatives | 540.63 |
| | | | **Other classes** | | | |
| **30** | 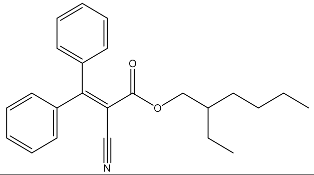 | 2-ethylhexyl 2-cyano-3,3-diphenylprop-2-enoate | Octocrylene (OC/ OCR) | 10% | Organic<br>UVB and UVA absorber | 361.49 |
| **31** | 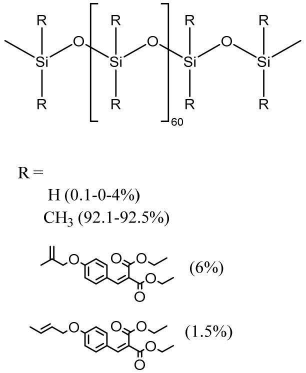 | Dimethicodiethylbenzalmalonate | Polysilicone-15 (PS-15) | 10% | Organic<br>UVB and UVA absorber | - |

| | | | | | | |
|---|---|---|---|---|---|---|
| **32** | 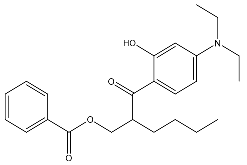 | hexyl 2-[4-(diethylamino)-2-hydroxybenzoyl]benzoate | Diethylamino hydroxybenzoyl hexyl benzoate (DHHB) | 10% | Organic UVA absorber | 397.52 |
| **33** | 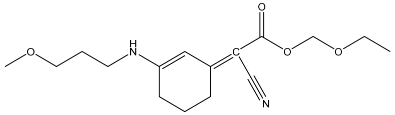 | 2-ethoxyethyl (2*Z*)-2-cyano-2-[3-(3-methoxypropylamino)cyclohex-2-en-1-ylidene]acetate | Methoxypropylamino Cyclohexenylidene Ethoxyethylcyanoacetate | 3% | Organic UVA and UVB absorber | 322.41 |
| | | **Inorganic UV Filters** | | | | |
| **34** | $Ti^{4+}$ $O^{2-}$ $O^{2-}$ | Titanium Dioxide/ dioxotitanium | Titanium dioxide and titanium dioxide $TiO_2$ (nano) | 25% | Inorganic UVB and UVA reflectors | 79.87 |
| **35** | $Zn^{2+}$ $O^{2-}$ | Zinc oxide/ oxozinc | Zinc oxide and zinc oxide (nano) (ZnO) | 25% | Inorganic UVB and UVA reflectors | 81.38 |

***Table S2.*** *Synthetic derivatives with photoprotective and antioxidant activities, as potential UV filters.*

| Number | Chemical structure | Activity | Values | Reference |
|---|---|---|---|---|
| **57** | 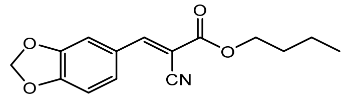 | Photoprotective | SPF = 1<br>$\lambda_c$ = 382 nm | [126] |
| **58** | 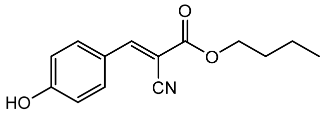 | | SPF = 2<br>$\lambda_c$ = 390 nm | |
| **59** | 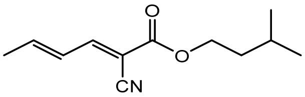 | | SPF = 5<br>$\lambda_c$ = 340 nm | |
| **60** | 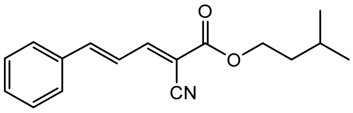 | | SPF = 4<br>$\lambda_c$ = 378 nm | |
| **61** | 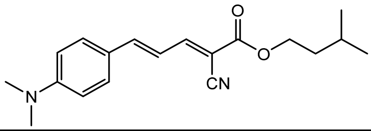 | | SPF = 3<br>$\lambda_c$ = 377 nm | |
| **62** | 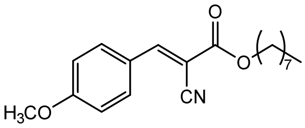 | | SPF = 5<br>$\lambda_c$ = 375 nm | |
| **63** | 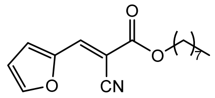 | | SPF = 2<br>$\lambda_c$ = 369 nm | |
| **64** | 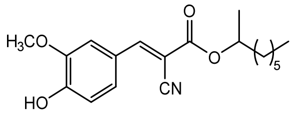 | | SPF = 2<br>$\lambda_c$ = 387 nm | |
| **65** | 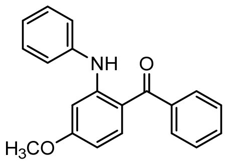 | Photoprotective | $\lambda_{max}$ = 275 nm | [127] |
| | | Phototoxicity | PIF = 0.916 – 1.281 (Non-phototoxic) | |
| **66** | 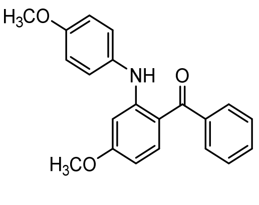 | Photoprotective | $\lambda_{max}$ = 285 nm | |
| | | Phototoxicity | PIF = 0.920 – 1.00 (Non-phototoxic) | |
| **67** | 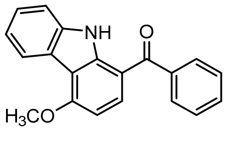 | Photoprotective | $\lambda_{max}$ = 292 nm | |
| | | Phototoxicity | PIF = 2.60 -2.80 (Phototoxic) | |
| **68** | | Photoprotective | $\lambda_{max}$ = 275 nm | |

| | | | | |
|---|---|---|---|---|
| | 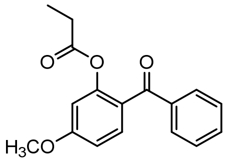 | Phototoxicity | PIF = 5.59 – 16.5 (Phototoxic) | |
| **69** | 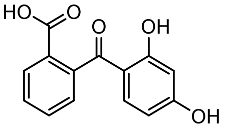 | Photoprotective | SPF = 16.20 at 3.0 x $10^{-2}$ mg/mL<br>$\lambda_{max}$ = 323 nm | [128] |
| **70** | 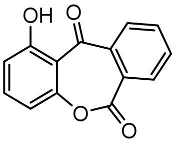 | Photoprotective | SPF = 7.63 at 3.0 x $10^{-2}$ mg/mL<br>$\lambda_{max}$ = 308 nm | |
| **71** | 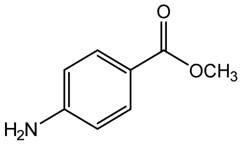 | Photoprotective | SPF = 20.60 | [129] |
| **72** | 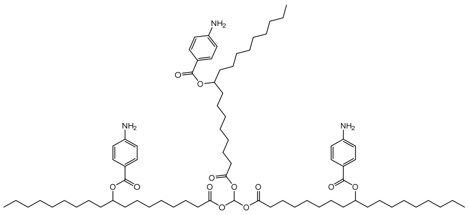 | Photoprotective | SPF = 26.17 | |
| **73** | 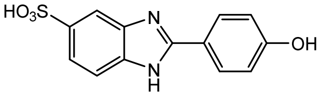 | Photoprotective | $\lambda_{max}$ = 308 nm<br>SPF = 4.74 ± 0.15 | [130] |
| | | Antioxidant (DPPH) | 2.04 ± 0.15 μmol Trolox/ mmol | |
| | | Phototoxicity (at 20 μM and UVA 20 J/cm$^2$ and UVB 0.5 J/cm$^2$) in % of viable cells | 77.3 ± 2.8% and 78.0 ± 3.5% | |
| **74** | 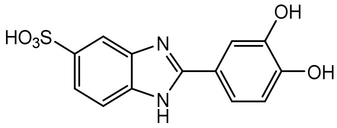 | Photoprotective | $\lambda_{max}$ = 313 nm<br>SPF = 9.83 ± 0.64 | |
| | | Antioxidant (DPPH) | 2145.31 ± 45.8 μmol Trolox/ mmol | |
| | | Phototoxicity (at 50 μM and UVA 20 J/cm$^2$ and UVB 0.5 J/cm$^2$) in % of viable cells | 77.4 ± 4.0% and 71.5 ± 2.5% | |
| | | Photostability (solar simulator for 1 hour before repeating HPLC analysis) | 94.9% | |

| | | | |
|---|---|---|---|
| **75** | 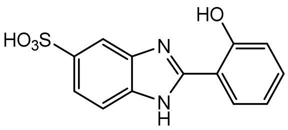 | Photoprotective | $\lambda_{max}$ = 315 nm<br>SPF = 2.22 ± 0.13 |
| | | Antioxidant (DPPH) | 2.03 ± 0.09 μmol Trolox/ mmol |
| | | Phototoxicity (at 50 μM and UVA 20 J/cm$^2$ and UVB 0.5 J/cm$^2$) in % of viable cells | 76.9 ± 2.0% and 74.3 ± 3.2% |
| **76** | 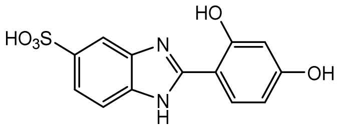 | Photoprotective | $\lambda_{max}$ = 318 nm<br>SPF = 2.18 ± 0.14 |
| | | Antioxidant (DPPH) | 21.41 ± 0.43 μmol Trolox/ mmol |
| | | Phototoxicity (at 50 μM and UVA 20 J/cm$^2$ and UVB 0.5 J/cm$^2$) in % of viable cells | 79.0 ± 6.0% and 70.8 ± 3.9% |
| **77** | 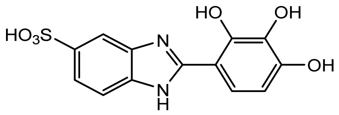 | Photoprotective | $\lambda_{max}$ = 328 nm<br>SPF = 5.99 ± 0.12 |
| | | Antioxidant (DPPH) | 1362.12 ± 133.96 μmol Trolox/ mmol |
| | | Phototoxicity (at 20 μM and UVA 20 J/cm$^2$ and UVB 0.5 J/cm$^2$) in % of viable cells | 18.4 ± 2.3% and 59.3 ± 3.4% |
| **78** | 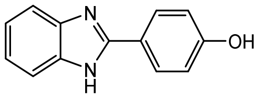 | Photoprotective | $\lambda_{max}$ = 306 nm |
| | | Antioxidant (DPPH) | 1.67 ± 0.08 μmol Trolox/ mmol |
| | | Phototoxicity (at 20 μM and UVA 20 J/cm$^2$ and UVB 0.5 J/cm$^2$) in % of viable cells | 72.8 ± 2.9% and 74.2 ± 1.3% |
| **79** | 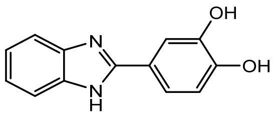 | Photoprotective | $\lambda_{max}$ = 311 nm<br>SPF = 5.20 ± 0.20 |
| | | Antioxidant (DPPH) | 1974.58 ± 16.89 μmol Trolox/ mmol |
| | | Phototoxicity (at 50 μM and UVA 20 J/cm$^2$ and UVB 0.5 J/cm$^2$) in % of viable cells | 62.9 ± 4.3% and 72.8 ± 1.0% |

| | | | |
|---|---|---|---|
| **80** | 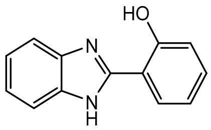 | Photoprotective | $\lambda_{max}$ = 315 nm<br>SPF = 3.57 ± 0.23 |
| | | Antioxidant (DPPH) | 16.47 ± 0.54 µmol Trolox/ mmol |
| | | Phototoxicity (at 50 µM and UVA 20 J/cm$^2$ and UVB 0.5 J/cm$^2$) in % of viable cells | 3.7 ± 0.5% and 1.1 ± 0.3% |
| **81** | 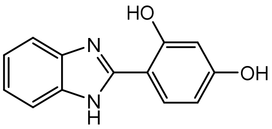 | Photoprotective | $\lambda_{max}$ = 315 nm<br>SPF = 1.96 ± 0.14 |
| | | Antioxidant (DPPH) | 0.87 ± 0.05 µmol Trolox/ mmol |
| | | Phototoxicity (at 50 µM and UVA 20 J/cm$^2$ and UVB 0.5 J/cm$^2$) in % of viable cells | 4.8 ± 0.6% and 19.8 ± 2.4% |
| **82** | 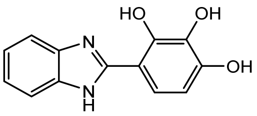 | Photoprotective | $\lambda_{max}$ = 325 nm<br>SPF = 2.85 ± 0.22 |
| | | Antioxidant (DPPH) | 1811.02 ± 61.7 µmol Trolox/ mmol |
| | | Phototoxicity (at 50 µM and UVA 20 J/cm$^2$ and UVB 0.5 J/cm$^2$) in % of viable cells | 1.2 ± 0.2% and 45.6 ± 2.6% |
| **83** | 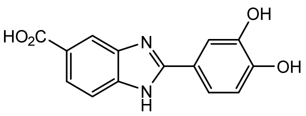 | Photoprotective | $\lambda_{max}$ = 317 nm<br>SPF = 2.21 ± 0.17 |
| | | Antioxidant (DPPH) | 1771.82 ± 84.75 µmol Trolox/ mmol |
| | | Phototoxicity (at 50 µM and UVA 20 J/cm$^2$ and UVB 0.5 J/cm$^2$) in % of viable cells | 74.3 ± 2.0% and 76.3 ± 3.6% |
| | | Photostability (solar simulator for 1 hour before repeating HPLC analysis) | 98.4% |

| | | | | |
|---|---|---|---|---|
| **84** | 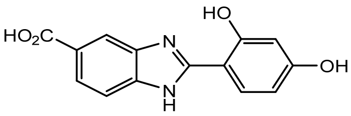 | Photoprotective | $\lambda_{max}$ = 321 nm<br>SPF = 2.85 ± 0.17 | |
| | | Antioxidant (DPPH) | 32.48 ± 2.38 µmol Trolox/mmol | |
| | | Phototoxicity (at 50 µM and UVA 20 J/cm$^2$ and UVB 0.5 J/cm$^2$) in % of viable cells | 80.7 ± 1.3% and 69.0 ± 0.2% | |
| **85** | 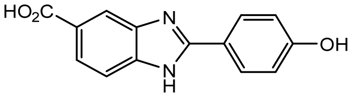 | Photoprotective | $\lambda_{max}$ = 312 nm<br>SPF = 6.01 ± 0.31 | |
| | | Antioxidant (DPPH) | 1.185 ± 0.02 µmol Trolox/mmol | |
| | | Phototoxicity (at 50 µM and UVA 20 J/cm$^2$ and UVB 0.5 J/cm$^2$) in % of viable cells | 76.7 ± 3.4% and 74.2 ± 3.4% | |
| **86** | 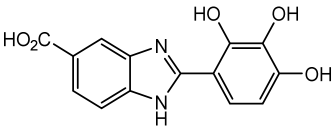 | Photoprotective | $\lambda_{max}$ = 332 nm<br>SPF = 3.69 ± 0.24 | |
| | | Antioxidant (DPPH) | 1241.03 ± 9.43 µmol Trolox/mmol | |
| | | Phototoxicity (at 50 µM and UVA 20 J/cm$^2$ and UVB 0.5 J/cm$^2$) in % of viable cells | 12.2 ± 1.3% and 60.1 ± 2.3% | |
| **87** | 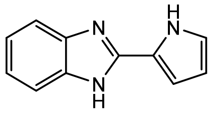 | Photoprotective (1-3% of the compound) | SPF = 2.44 – 3.06<br>$\lambda_c$ = 341 - 364 nm | |
| | | Antioxidant (DPPH) | % inhibition = 70.00 ± 3.25 | |
| **88** | 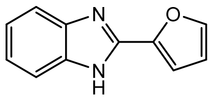 | Photoprotective (1-3% of the compound) | SPF = 6.84 – 11.15<br>$\lambda_c$ = 368 - 371 nm | [131] |
| | | Antioxidant (DPPH) | % inhibition = 15.46 ± 0.98 | |

| | | | | |
|---|---|---|---|---|
| **89** | 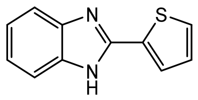 | Photoprotective (1-3% of the compound) | SPF = 2.41 - 2.79<br>$\lambda_c$ = 340 – 344 nm | |
| | | Antioxidant (DPPH) | % inhibition = 35.05 ± 0.87 | |
| **90** | 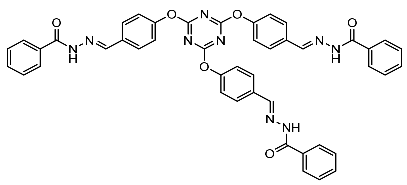 | Photoprotective | SPF = 9.0 ± 0.1<br>$\lambda_c$ = 371 nm | |
| | | Antioxidant (DPPH) at 400 μM | % inhibition = 2.4 ± 0.7 | |
| **91** | 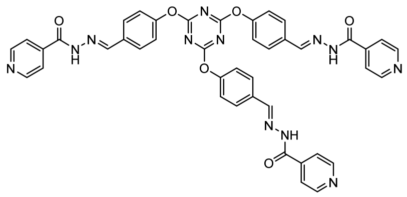 | Photoprotective | SPF = 5.0 ± 0.2<br>$\lambda_c$ = 365 nm | |
| | | Antioxidant (DPPH) at 400 μM | % inhibition = 6.1 ± 0.5 | |
| **92** | 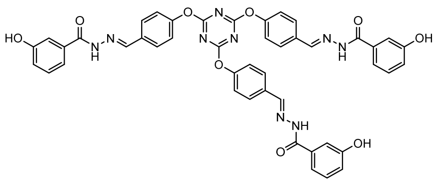 | Photoprotective | SPF = 17.0 ± 0.6<br>$\lambda_c$ = 371 nm | |
| | | Antioxidant (DPPH) at 400 μM | % inhibition = 0.8 ± 0.3 | [25] |
| **93** | 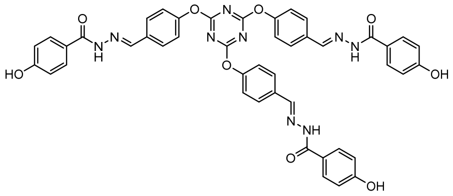 | Photoprotective | SPF = 4.0 ± 1.7<br>$\lambda_c$ = 356 nm | |
| | | Antioxidant (DPPH) at 400 μM | % inhibition = 6.3 ± 0.7 | |
| **94** | 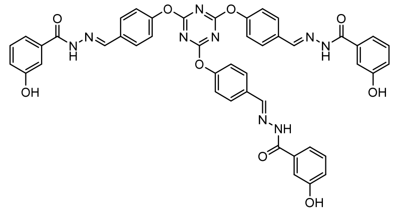 | Photoprotective | SPF = 7.0 ± 0.8<br>$\lambda_c$ = 363 nm | |
| | | Antioxidant (DPPH) at 400 μM | % inhibition = 8.2 ± 1.6 | |
| **95** | 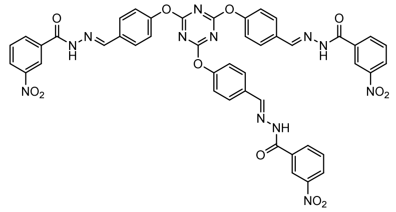 | Photoprotective | SPF = 4.0 ± 0.2<br>$\lambda_c$ = 383 nm | |
| | | Antioxidant (DPPH) at 400 μM | % inhibition = 3.5 ± 0.4 | |

| | | | | |
|---|---|---|---|---|
| **96** | 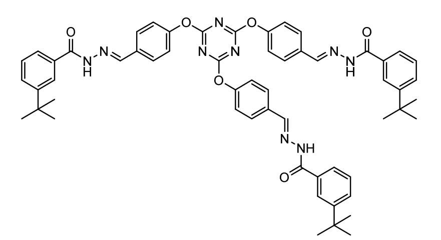 | Photoprotective | SPF = 14.0 ± 1.7<br>$\lambda_c$ = 360 nm | |
| | | Antioxidant (DPPH) at 400 μM | % inhibition = 5.9 ± 0.6 | |
| **97** | 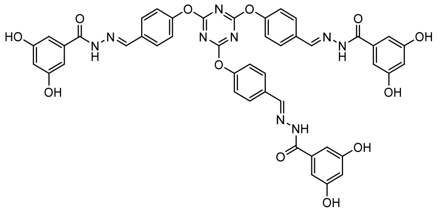 | Photoprotective | SPF = 3.0 ± 0.2<br>$\lambda_c$ = 382 nm | |
| | | Antioxidant (DPPH) at 400 μM | % inhibition = 75.6 ± 0.2 | |
| **98** | 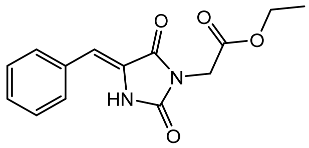 | Photoprotective | SPF = 4.79<br>$\lambda_c$ = 339 nm | |
| **99** | 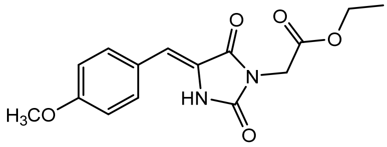 | Photoprotective | SPF = 3.20<br>$\lambda_c$ = 363 nm | |
| **100** | 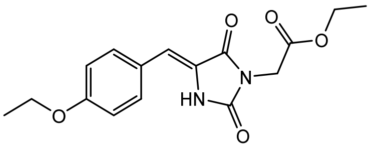 | Photoprotective | SPF = 3.16<br>$\lambda_c$ = 367 nm | [132] |
| **101** | 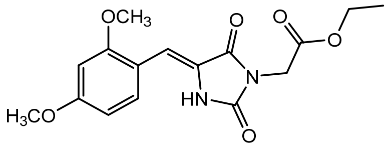 | Photoprotective | SPF = 2.00<br>$\lambda_c$ = 386 nm | |
| | | Cytotoxicity (human keratinocytes (K) and human fibroblasts (F)) | % cell viability (K) = 93<br>% cell viability (F) =105 | |
| **102** | 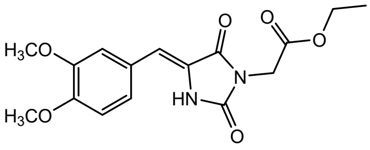 | Photoprotective | SPF = 2.50<br>$\lambda_c$ = 379 nm | |
| | | Cytotoxicity (human keratinocytes (K) and human fibroblasts (F)) | % cell viability (K) = 98<br>% cell viability (F) = 89 | |

| | | | |
|---|---|---|---|
| **103** | 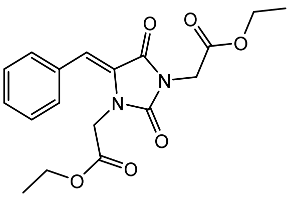 | Photoprotective | SPF = 2.97<br>$\lambda_c$ = 331 nm |
| **104** | 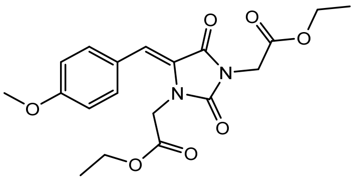 | Photoprotective | SPF = 3.07<br>$\lambda_c$ = 366 nm |
| | | Cytotoxicity (human keratinocytes (K) and human fibroblasts (F)) | % cell viability (K) = 95<br>% cell viability (F) = 107 |
| **105** | 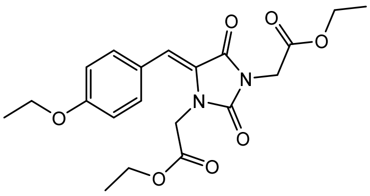 | Photoprotective | SPF = 2.49<br>$\lambda_c$ = = 370 nm |
| **106** | 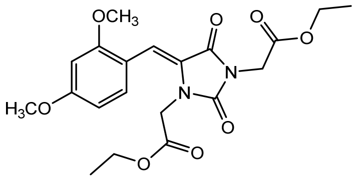 | Photoprotective | SPF = 1.84<br>$\lambda_c$ = 383 nm |
| **107** | 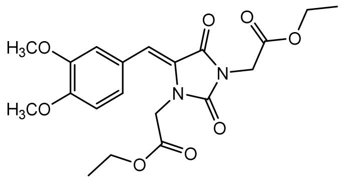 | Photoprotective | SPF = 1.87<br>$\lambda_c$ = 382 nm |
| **108** | 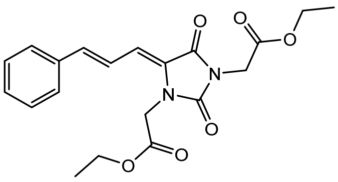 | Photoprotective | SPF = 1.87<br>$\lambda_c$ = 381 nm |
| | | Cytotoxicity (human keratinocytes (K) and human fibroblasts (F)) | % cell viability (K) = 100<br>% cell viability (F) =94 |
| **109** | 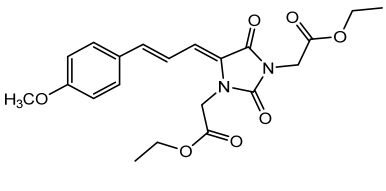 | Photoprotective | SPF = 1.68<br>$\lambda_c$ = 391 nm |
| | | Cytotoxicity (human keratinocytes (K) and human fibroblasts (F)) | % cell viability (K) = 100<br>% cell viability (F) =97 |

| | | | | |
|---|---|---|---|---|
| **110** | 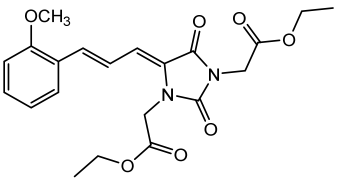 | Photoprotective | SPF = 1.94<br>$\lambda_c$ = 389 nm | |
| **111** | 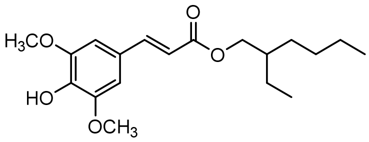 | Photoprotective (after UV irradiation of 8.32 W/m² for 1 hour) | LoA = 18% | |
| | | Antioxidant (DPPH) | $IC_{50}$ = 22.2 ± 1.1 nmol | |
| **112** | 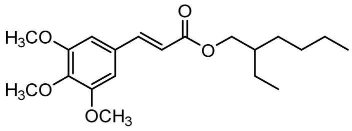 | Photoprotective (after UV irradiation of 8.32 W/m² for 1 hour) | LoA = 22% | |
| | | Antioxidant (DPPH) | - | |
| **113** | 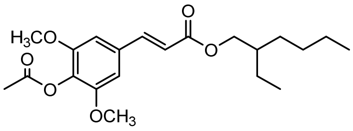 | Photoprotective (after UV irradiation of 8.32 W/m² for 1 hour) | LoA = 31% | |
| | | Antioxidant (DPPH) | - | |
| **114** | 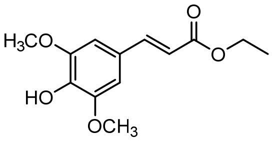 | Photoprotective (after UV irradiation of 8.32 W/m² for 1 hour) | LoA = 26% | [133] |
| | | Antioxidant (DPPH) | $IC_{50}$ = 18.0 ± 0.9 nmol | |
| **115** | 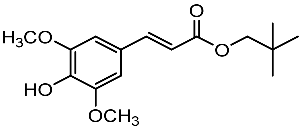 | Photoprotective (after UV irradiation of 8.32 W/m² for 1 hour) | LoA = 35% | |
| | | Antioxidant (DPPH) | $IC_{50}$ = 17.0 ± 0.7 nmol | |
| **116** | 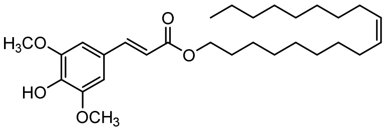 | Photoprotective (after UV irradiation of 8.32 W/m² for 1 hour) | LoA = 21% | |
| | | Antioxidant (DPPH) | $IC_{50}$ = 19.9 ± 1.0 nmol | |
| **117** | 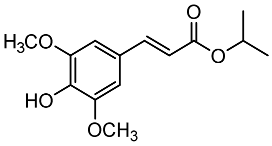 | Photoprotective (after UV irradiation of 8.32 W/m² for 1 hour) | LoA = 52% | |
| | | Antioxidant (DPPH) | $IC_{50}$ = 14.4 ± 0.4 nmol | |

| | | | |
|---|---|---|---|
| **118** | 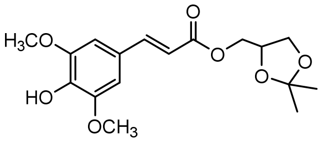 | Photoprotective (after UV irradiation of 8.32 W/m² for 1 hour) | LoA = 13% |
| | | Antioxidant (DPPH) | $IC_{50}$ = 19.8 ± 1.0 nmol |
| **119** | 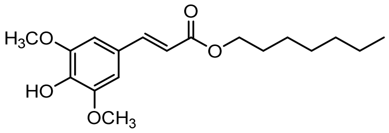 | Photoprotective (after UV irradiation of 8.32 W/m² for 1 hour) | LoA = 26% |
| | | Antioxidant (DPPH) | $IC_{50}$ = 20.9 ± 1.1 nmol |
| **120** | 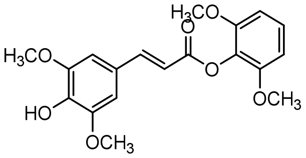 | Photoprotective (after UV irradiation of 8.32 W/m² for 1 hour) | LoA = 73% |
| | | Antioxidant (DPPH) | $IC_{50}$ = 21.1 ± 1.1 nmol |
| **121** | 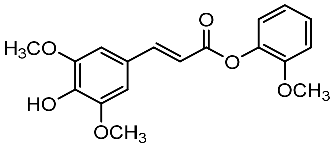 | Photoprotective (after UV irradiation of 8.32 W/m² for 1 hour) | LoA = 67% |
| | | Antioxidant (DPPH) | $IC_{50}$ = 17.1 ± 0.8 nmol |
| **122** | 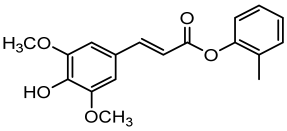 | Photoprotective (after UV irradiation of 8.32 W/m² for 1 hour) | LoA = 70% |
| | | Antioxidant (DPPH) | $IC_{50}$ = 15.3 ± 0.5 nmol |
| **123** | 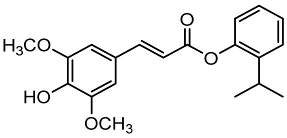 | Photoprotective (after UV irradiation of 8.32 W/m² for 1 hour) | LoA = 66% |
| | | Antioxidant (DPPH) | $IC_{50}$ = 13.7 ± 0.6 nmol |
| **124** | 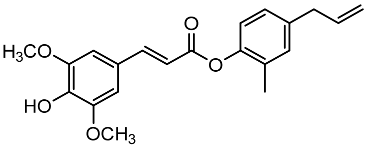 | Photoprotective (after UV irradiation of 8.32 W/m² for 1 hour) | LoA = 85% |
| | | Antioxidant (DPPH) | $IC_{50}$ = 19.4 ± 0.9 nmol |
| **125** | 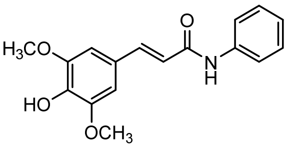 | Photoprotective (after UV irradiation of 8.32 W/m² for 1 hour) | LoA = 20% |
| | | Antioxidant (DPPH) | $IC_{50}$ = 8.9 ± 0.3 nmol |

| | | | | |
|---|---|---|---|---|
| **126** | 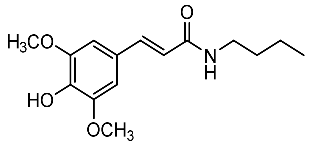 | Photoprotective (after UV irradiation of 8.32 W/m$^2$ for 1 hour) | LoA = 40% | |
| | | Antioxidant (DPPH) | $IC_{50}$ = 15.9 ± 0.7 nmol | |
| **127** | 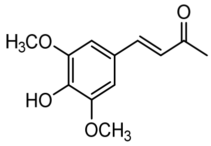 | Photoprotective (after UV irradiation of 8.32 W/m$^2$ for 1 hour) | LoA = 45% | |
| | | Antioxidant (DPPH) | $IC_{50}$ = 9.4 ± 0.4 nmol | |
| **128** | 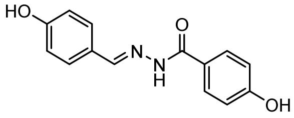 | Antioxidant (DPPH) | $IC_{50}$ = 275 μM | |
| | | Photoprotective | SPF = 3<br>$\lambda_c$ = 376 nm | |
| **129** | 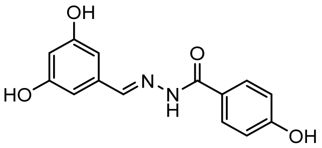 | Photoprotective | SPF = 5<br>$\lambda_c$ = 368 nm | |
| **130** | 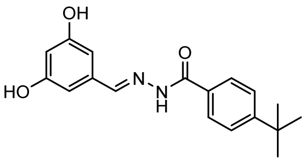 | Photoprotective | SPF = 4<br>$\lambda_c$ = 366 nm | |
| **131** | 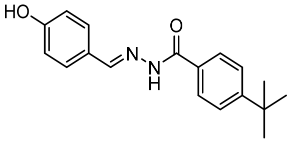 | Photoprotective | SPF = 2<br>$\lambda_c$ = 379 nm | [134] |
| **132** | 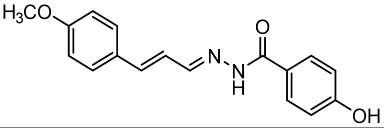 | Photoprotective | SPF = 2<br>$\lambda_c$ = 388 nm | |
| **133** | 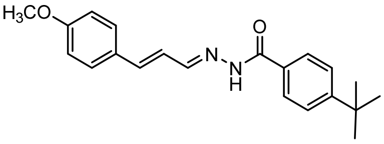 | Photoprotective | SPF = 4<br>$\lambda_c$ = 386 nm | |
| **134** | 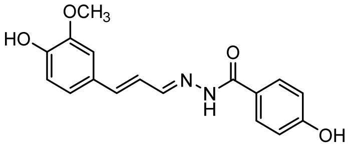 | Antioxidant (DPPH) | $IC_{50}$ = 88.2 μM | |
| | | Photoprotective | SPF = 2<br>$\lambda_c$ = 389 nm | |

| | | | | |
|---|---|---|---|---|
| **135** | 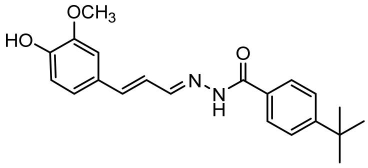 | Antioxidant (DPPH) | $IC_{50}$ = 109.6 μM | |
| | | Photoprotective | SPF = 2<br>$\lambda_c$ = 387 nm | |
| **136** | 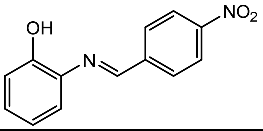 | Photoprotective | SPF = 2.0 ± 0.5 | [135] |
| **137** | 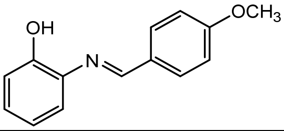 | Photoprotective | SPF = 10.0 ± 0.2<br>$\lambda_c$ = 383 nm | |
| **138** | 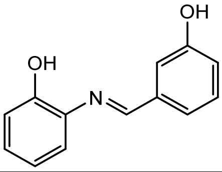 | Photoprotective | SPF = 6.0 ± 0.7<br>$\lambda_c$ = 389 nm | |
| **139** | 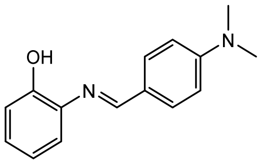 | Photoprotective | SPF = 5.0 ± 0.3<br>$\lambda_c$ = 388 nm | |
| **140** | 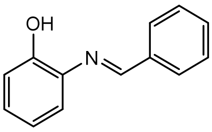 | Photoprotective | SPF = 6.0 ± 1.1<br>$\lambda_c$ = 387 nm | |
| **141** | 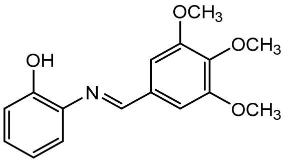 | Photoprotective | SPF = 6.0 ± 1.1<br>$\lambda_c$ = 387 nm | |
| **142** | 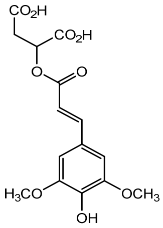 | Photoprotective (after UV irradiation of 8.32 W/m² for 1 hour) | LoA = 19.1% | [24] |
| | | Antioxidant (DPPH) | $IC_{50}$ = 7.9 nmol | |
| **143** | 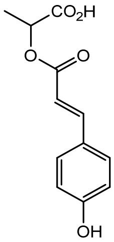 | Photoprotective (after UV irradiation of 8.32 W/m² for 1 hour) | LoA = 21.9% | |
| | | Antioxidant (DPPH) | - | |

| | | | |
|---|---|---|---|
| **144** | 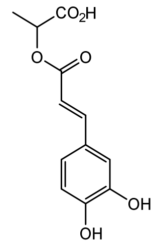 | Photoprotective (after UV irradiation of 8.32 W/m$^2$ for 1 hour) | LoA = 20.2% |
| | | Antioxidant (DPPH) | $IC_{50}$ = 9.9 nmol |
| **145** | 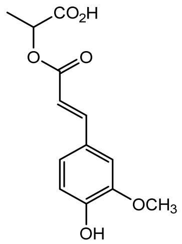 | Photoprotective (after UV irradiation of 8.32 W/m$^2$ for 1 hour) | LoA = 24.4% |
| | | Antioxidant (DPPH) | $IC_{50}$ = 13.9 nmol |
| **146** | 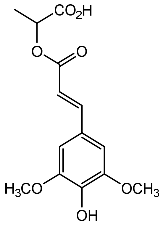 | Photoprotective (after UV irradiation of 8.32 W/m$^2$ for 1 hour) | LoA = 26.1% |
| | | Antioxidant (DPPH) | $IC_{50}$ = 10.6 nmol |
| **147** | 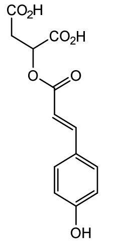 | Photoprotective (after UV irradiation of 8.32 W/m$^2$ for 1 hour) | LoA = 22.5% |
| | | Antioxidant (DPPH) | - |
| **148** | 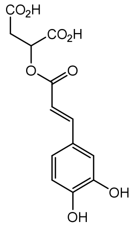 | Photoprotective (after UV irradiation of 8.32 W/m$^2$ for 1 hour) | LoA = 31.5% |
| | | Antioxidant (DPPH) | $IC_{50}$ = 6.8 nmol |
| **149** | 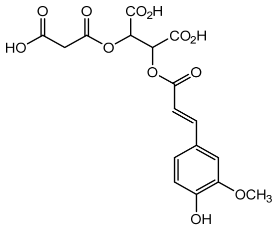 | Photoprotective (after UV irradiation of 8.32 W/m$^2$ for 1 hour) | LoA = 26.3% |
| | | Antioxidant (DPPH) | $IC_{50}$ = 10.2 nmol |
| **150** | 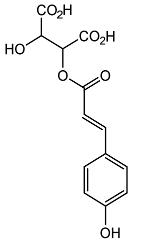 | Photoprotective (after UV irradiation of 8.32 W/m$^2$ for 1 hour) | LoA = 20.2% |
| | | Antioxidant (DPPH) | - |

| | | | |
|---|---|---|---|
| **151** | 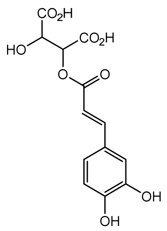 | Photoprotective (after UV irradiation of 8.32 W/m[2] for 1 hour) | LoA = 16.1% |
| | | Antioxidant (DPPH) | $IC_{50}$ = 5.2 nmol |
| **152** | 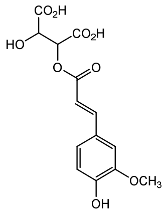 | Photoprotective (after UV irradiation of 8.32 W/m[2] for 1 hour) | LoA = 23.2% |
| | | Antioxidant (DPPH) | $IC_{50}$ = 11.9 nmol |
| **153** | 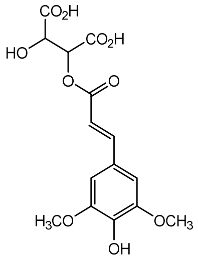 | Photoprotective (after UV irradiation of 8.32 W/m[2] for 1 hour) | LoA = 24.3% |
| | | Antioxidant (DPPH) | $IC_{50}$ = 6.9 nmol |
| **154** | 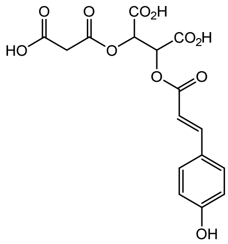 | Photoprotective (after UV irradiation of 8.32 W/m[2] for 1 hour) | LoA = 15.7% |
| | | Antioxidant (DPPH) | - |
| **155** | 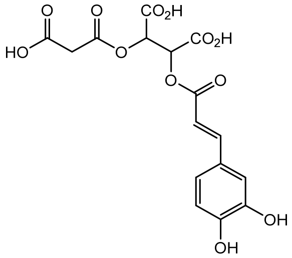 | Photoprotective (after UV irradiation of 8.32 W/m[2] for 1 hour) | LoA = 15.5% |
| | | Antioxidant (DPPH) | $IC_{50}$ = 3.9 nmol |
| **156** | 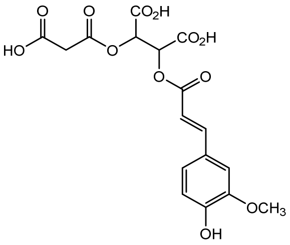 | Photoprotective (after UV irradiation of 8.32 W/m[2] for 1 hour) | LoA = 14.3% |
| | | Antioxidant (DPPH) | $IC_{50}$ =5.5 nmol |
| **157** | 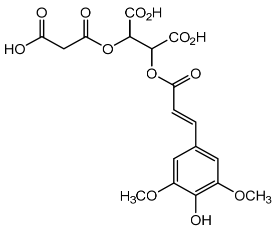 | Photoprotective (after UV irradiation of 8.32 W/m[2] for 1 hour) | LoA = 17.4% |
| | | Antioxidant (DPPH) | $EC_{50}$ = 4.2 nmol |

| | | | | |
|---|---|---|---|---|
| **158** | 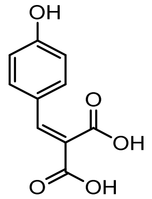 | Photoprotective (after UV irradiation of 8.32 W/m² for 1 hour) | LoA = 3.0% $\lambda_{max}$ = 314 nm | [137] |
| | | Antioxidant (DPPH) | - | |
| **159** | 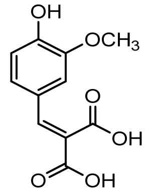 | Photoprotective (after UV irradiation of 8.32 W/m² for 1 hour) | LoA = 1.9% $\lambda_{Max}$ = 328 nm | |
| | | Antioxidant (DPPH) | $IC_{50}$ (nmol) = 20.7 | |
| **160** | 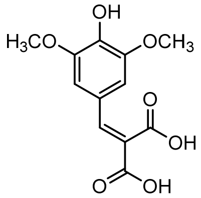 | Photoprotective (after UV irradiation of 8.32 W/m² for 1 hour) | LoA = 8.9% $\lambda_{Max}$ = 330 nm | |
| | | Antioxidant (DPPH) | $IC_{50}$ (nmol) = 3.9 | |
| **161** | 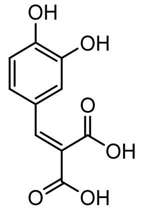 | Photoprotective | LoA = 4.9% $\lambda_{Max}$ = 340 nm | |
| | | Antioxidant (DPPH) | $IC_{50}$ (nmol) = 3.0 | |
| **162** | 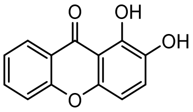 | Phototoxicity | Cell viability = 100% (at 200 μM in non-radiated and irradiated HaCaT keratinocytes) | [138] |
| | | Antioxidant (DPPH) | $IC_{50}$ = 28.4 ± 0.2 μM | |
| **163** | 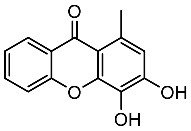 | Antioxidant (DPPH) | $IC_{50}$ = 31.2 ± 4.8 μM | |
| **164** | 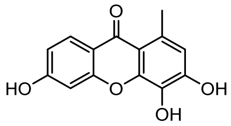 | Antioxidant (DPPH) | $IC_{50}$ = 47.3 ± 0.4 μM | |
| **165** | 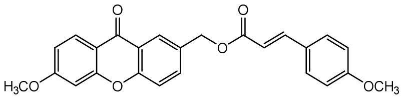 | Photoprotective (1 hour after irradiation with solar light simulator conducted at 500 W/m²) | $\lambda_{max}$ = 303 nm $\lambda_c$ = 345 SPF = 14.69 Decrease of SPF after irradiation 10.97% | [26] |

| | | | | |
|---|---|---|---|---|
| **166** | 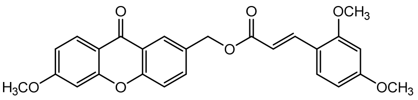 | Photoprotective (1 hour after irradiation with solar light simulator conducted at 500 W/m[2]) | $\lambda_{max}$ = 299, 329 nm<br>$\lambda_c$ = 381<br>SPF = 19.69<br>SPF after irradiation = 17.25% | |
| | | Phototoxicity (human keratinocytes (K) HaCaT and skin fibroblasts (F)) at concentration of 50 μM | % cell viability (K) = 82<br>% cell viability (F) = 80 | |
| **167** | 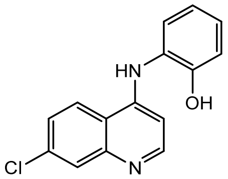 | Photoprotective | SPF = 4.0 ± 0.9<br>$\lambda_c$ =387 nm | |
| **168** | 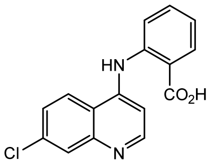 | Photoprotective | SPF = 3.0 ± 0.5<br>$\lambda_c$ = 389 nm | |
| **169** | 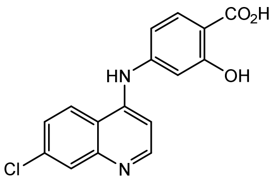 | Photoprotective | SPF = 3.0 ± 0.1<br>$\lambda_c$ = 388 nm | |
| **170** | 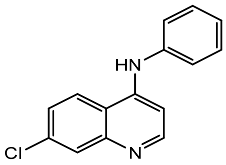 | Photoprotective | SPF = 4.0 ± 0.0<br>$\lambda_c$ = 385 nm | [139] |
| **171** | 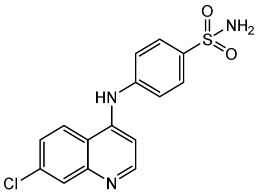 | Photoprotective | SPF = 3.0 ± 0.2<br>$\lambda_c$ = 388 nm | |
| **172** | 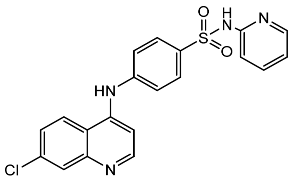 | Photoprotective | SPF = 2.0 ± 0.3<br>$\lambda_c$ = 388 nm nm | |
| **173** | 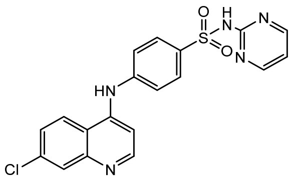 | Photoprotective | SPF = 2.0 ± 0.1<br>$\lambda_c$ = 386 nm | |

| | | | | |
|---|---|---|---|---|
| **174** | 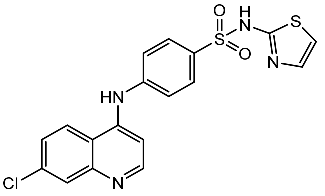 | Photoprotective | SPF = 4.0 ± 0.4<br>$\lambda_c$ = 385 nm | |
| **175** | 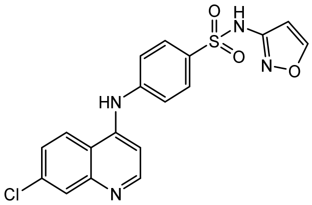 | Photoprotective | SPF = 4.0 ± 0.0<br>$\lambda_c$ = 381 nm | |
| **176** | 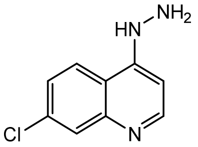 | Photoprotective | SPF = 11.0 ± 0.1<br>$\lambda_c$ = 376 nm | |
| **177** | 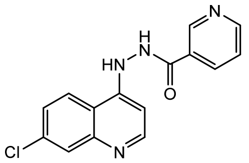 | Photoprotective | SPF = 6.0 ± 0.3<br>$\lambda_c$ = 377 nm | |
| **178** | 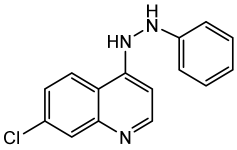 | Photoprotective | SPF = 3.0 ± 0.4<br>$\lambda_c$ = 384 nm | |
| **179** | 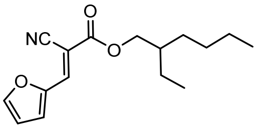 | Photoprotective | $\lambda_{max}$ = 339 nm<br>SPF = 17.30 (with 5% of the compound in formulation) | [140] |
| **180** | 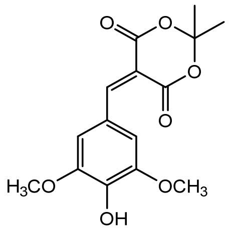 | Photoprotective (after UV irradiation of 8.32 W/m$^2$ for 1 hour) | LoA = 11.4% | [141] |
| | | Antioxidant (DPPH) | $IC_{50}$ = 19.70 ± 1.00 mM | |
| | | Tyrosinase inhibition | $IC_{50}$ = 17 ± 2 μM | |
| **181** | 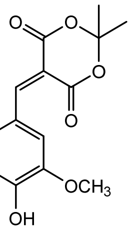 | Photoprotective (after UV irradiation of 8.32 W/m$^2$ for 1 hour) | LoA = 9.0% | |
| | | Antioxidant (DPPH) | $IC_{50}$ > 400 mM | |
| | | Tyrosinase inhibition | $IC_{50}$ = 23 ± 3 μM | |

| | | | |
|---|---|---|---|
| **182** | 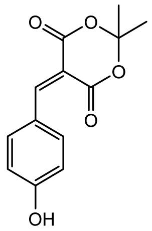 | Photoprotective (after UV irradiation of 8.32 W/m$^2$ for 1 hour) | LoA = 3.5% |
| | | Antioxidant (DPPH) | $IC_{50}$ > 400 mM |
| | | Tyrosinase inhibition | $IC_{50}$ = 106 ± 13 μM |
| **183** | 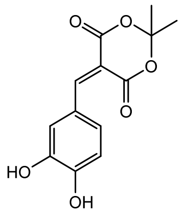 | Photoprotective (after UV irradiation of 8.32 W/m$^2$ for 1 hour) | LoA = 6.4% |
| | | Antioxidant (DPPH) | $IC_{50}$ = 16.70 ± 0.80 mM |
| | | Tyrosinase inhibition | $IC_{50}$ = 13 ± 2 μM |
| **184** | 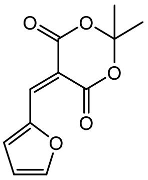 | Photoprotective (after UV irradiation of 8.32 W/m$^2$ for 1 hour) | LoA = 3.5% |
| | | Antioxidant (DPPH) | $IC_{50}$ > 400 mM |
| | | Tyrosinase inhibition | $IC_{50}$ = 465 ± 56 μM |
| **185** | 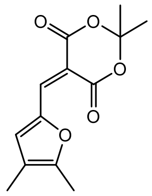 | Photoprotective (after UV irradiation of 8.32 W/m$^2$ for 1 hour) | LoA = 4.2% |
| | | Antioxidant (DPPH) | - |
| | | Tyrosinase inhibition | - |
| **186** | 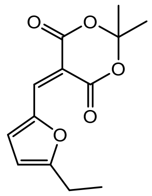 | Photoprotective (after UV irradiation of 8.32 W/m$^2$ for 1 hour) | LoA = 5.1% |
| | | Antioxidant (DPPH) | - |
| | | Tyrosinase inhibition | - |
| **187** | 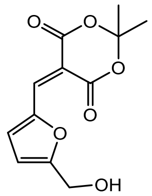 | Photoprotective (after UV irradiation of 8.32 W/m$^2$ for 1 hour) | LoA = 3.4% |
| | | Antioxidant (DPPH) | $IC_{50}$ > 400 mM |
| | | Tyrosinase inhibition | - |

| | | | |
|---|---|---|---|
| **188** | 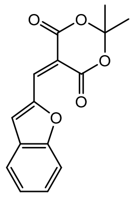 | Photoprotective (after UV irradiation of 8.32 W/m$^2$ for 1 hour) | LoA = 28.7% |
| | | Antioxidant (DPPH) | - |
| | | Tyrosinase inhibition | $IC_{50}$ = 252 ± 30 μM |
| **189** | 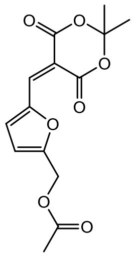 | Photoprotective (after UV irradiation of 8.32 W/m$^2$ for 1 hour) | LoA = 12.9% |
| | | Antioxidant (DPPH) | - |
| | | Tyrosinase inhibition | - |
| **190** | 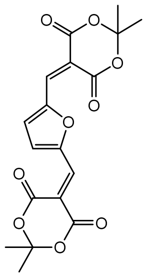 | Photoprotective (after UV irradiation of 8.32 W/m$^2$ for 1 hour) | LoA = 12.4% |
| | | Antioxidant (DPPH) | $IC_{50}$ > 400 mM |
| | | Tyrosinase inhibition | - |
| **191** | 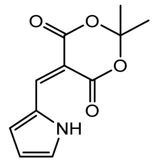 | Photoprotective (after UV irradiation of 8.32 W/m$^2$ for 1 hour) | LoA = 0.1% |
| | | Antioxidant (DPPH) | - |
| | | Tyrosinase inhibition | - |
| **192** | 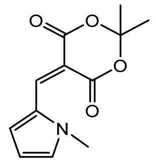 | Photoprotective (after UV irradiation of 8.32 W/m$^2$ for 1 hour) | LoA = 3.5% |
| | | Antioxidant (DPPH) | - |
| | | Tyrosinase inhibition | - |
| **193** | 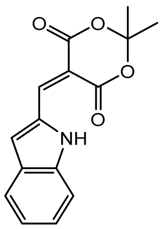 | Photoprotective (after UV irradiation of 8.32 W/m$^2$ for 1 hour) | LoA = 11.9% |
| | | Antioxidant (DPPH) | - |
| | | Tyrosinase inhibition | - |

## References:


1. Jussila, A.; Huotari-Orava, R.; Ylianttila, L.; Partonen, T.; Snellman, E., Narrow-band ultraviolet b radiation induces the expression of beta-endorphin in human skin in vivo. *J Photochem Photobiol B* **2016,** *155*, 104-108, 10.1016/j.jphotobiol.2016.01.007.
2. Dahmane, R.; Pandel, R.; Trebse, P.; Poljsak, B., The role of sun exposure in skin aging. In *Sun exposure: Risk factors, protection practices and health effects*, Nova Science Publishers, Inc.: 2015; pp 1-40.
3. Labille, J.; Catalano, R.; Slomberg, D.; Motellier, S.; Pinsino, A.; Hennebert, P.; Santaella, C.; Bartolomei, V., Assessing sunscreen lifecycle to minimize environmental risk posed by nanoparticulate uv-filters – a review for safer-by-design products. *Front Environ Sci* **2020,** *8*, 10.3389/fenvs.2020.00101.
4. Ma, Y.; Yoo, J., History of sunscreen: An updated view. *J Cosmetol Dermatol* **2021,** *20*, 1044-1049, 10.1111/jocd.14004.
5. Pawlowski, S.; Petersen-Thiery, M. Sustainable sunscreens: A challenge between performance, animal testing ban, and human and environmental safety. In *Handbook of Environmental Chemistry*, Springer: 2020; Vol. 94, pp 185-207.
6. Abiola, T. T.; Whittock, A. L.; Stavros, V. G., Unravelling the photoprotective mechanisms of nature-inspired ultraviolet filters using ultrafast spectroscopy. *Molecules* **2020,** *25*, 10.3390/molecules25173945.
7. L´Oreal. Garnier ambre solaire. 2020.
8. Roelandts, R., Vanhee, J., Bonamie, A., Kerkhofs, L., Degreef, H., A survey of ultraviolet absorbers in commercially available sun products. *Int J Dermatol* **1983,** *22*, 247-255.
9. Regulation (ec) nº 1223/2009 of the european parliament and of the council: Current consolidated version (01/10/2021).
10. Bissonnette, R. Update on sunscreens.
11. Chen, L. L.; Tooley, I.; Wang, S. Q., Nanotechnology in photoprotection. In *Nanotechnology in dermatology*, Springer New York: 2013; Vol. 9781461450344, pp 9-18.
12. Valle-Sistac, J.; Molins-Delgado, D.; Díaz, M.; Ibáñez, L.; Barceló, D.; Silvia Díaz-Cruz, M., Determination of parabens and benzophenone-type uv filters in human placenta: First description of the existence of benzyl paraben and benzophenone-4. *Environ Int* **2016,** *88*, 243-249, 10.1016/j.envint.2015.12.034.
13. Rehfeld, A.; Egeberg, D. L.; Almstrup, K.; Petersen, J. H.; Dissing, S.; Skakkebæk, N. E., Edc impact: Chemical uv filters can affect human sperm function in a progesterone-like manner. *Endocr Connect* **2018,** *7*, 16-25, 10.1530/EC-17-0156.
14. Bachelot, M.; Li, Z.; Munaron, D.; Le Gall, P.; Casellas, C.; Fenet, H.; Gomez, E., Organic uv filter concentrations in marine mussels from french coastal regions. *Sci Total Environ* **2012,** *420*, 273-279, 10.1016/j.scitotenv.2011.12.051.
15. He, H.; Li, A.; Li, S.; Tang, J.; Li, L.; Xiong, L., Natural components in sunscreens: Topical formulations with sun protection factor (spf). *Biomed Pharmacother* **2021,** *134*, 10.1016/j.biopha.2020.111161.
16. Giraldo, A.; Montes, R.; Rodil, R.; Quintana, J. B.; Vidal-Liñán, L.; Beiras, R., Ecotoxicological evaluation of the uv filters ethylhexyl dimethyl p-aminobenzoic acid and octocrylene using marine organisms isochrysis galbana, mytilus galloprovincialis and paracentrotus lividus. *Arch Environ Contam Toxicol* **2017,** *72*, 606-611, 10.1007/s00244-017-0399-4.
17. Lozano, C.; Givens, J.; Stien, D.; Matallana-Surget, S.; Lebaron, P. Bioaccumulation and toxicological effects of uv-filters on marine species. In *Handbook of Environmental Chemistry*, Springer: 2020; Vol. 94, pp 85-130.
18. Bahia, M. F., *Proteção solar actualização*. 1st ed.; Editora da Universidade do Porto: 2003.
19. Stiefel, C., Schwack, W., Reactions of cosmetic uv filters with skin proteins: Model studies of esters with primary amines. *Trends Photochem Photobiol* **2013,** *15*, 105-116.
20. Kockler, J.; Oelgemöller, M.; Robertson, S.; Glass, B. D., Photostability of sunscreens. *J Photochem Photobiol C Photochem Rev* **2012,** *13*, 91-110, 10.1016/j.jphotochemrev.2011.12.001.
21. Cefali, L. C.; Ataide, J. A.; Moriel, P.; Foglio, M. A.; Mazzola, P. G., Plant-based active photoprotectants for sunscreens. *Int J Cosmet Sci* **2016,** *38*, 346-353, 10.1111/ics.12316.

22. Vega, J.; Bonomi-Barufi, J.; Gomez-Pinchetti, J. L.; Figueroa, F. L., Cyanobacteria and red macroalgae as potential sources of antioxidants and uv radiation-absorbing compounds for cosmeceutical applications. *Mar Drugs* **2020,** *18*, 10.3390/md18120659.
23. Solano, F., Photoprotection and skin pigmentation: Melanin-related molecules and some other new agents obtained from natural sources. *Molecules* **2020,** *25*, 10.3390/molecules25071537.
24. Peyrot, C.; Mention, M. M.; Fournier, R.; Brunissen, F.; Couvreur, J.; Balaguer, P.; Allais, F., Expeditious and sustainable two-step synthesis of sinapoyl-l-malate and analogues: Towards non-endocrine disruptive bio-based and water-soluble bioactive compounds. *Green Chem* **2020,** *22*, 6510-6518, 10.1039/d0gc02763d.
25. Reis, J. S.; Corrêa, M. A.; Ribeiro, C. A.; Dos Santos, J. L., Synthesis and evaluation of 1,3,5-triazine derivatives as sunscreens useful to prevent skin cancer. *Bioorg Med Chem Lett* **2019,** *29*, 10.1016/j.bmcl.2019.126755.
26. Popiół, J.; Gunia-Krzyżak, A.; Słoczyńska, K.; Koczurkiewicz-Adamczyk, P.; Piska, K.; Wójcik-Pszczoła, K.; Żelaszczyk, D.; Krupa, A.; Żmudzki, P.; Marona, H., *et al.*, The involvement of xanthone and (e)-cinnamoyl chromophores for the design and synthesis of novel sunscreening agents. *Int J Mol Sci* **2021,** *22*, 1-23, 10.3390/ijms22010034.
27. Herzog, B.; Wehrle, M.; Quass, K., Photostability of uv absorber systems in sunscreens. *Photochem Photobiol* **2009,** *85*, 869-878, 10.1111/j.1751-1097.2009.00544.x.
28. Bonda, C. A.; Lott, D., Sunscreen photostability. In *Principles and practice of photoprotection*, Springer International Publishing: 2016; pp 247-273.
29. Afonso, S.; Horita, K.; Sousa E Silva, J. P.; Almeida, I. F.; Amaral, M. H.; Lobão, P. A.; Costa, P. C.; Miranda, M. S.; Esteves Da Silva, J. C. G.; Sousa Lobo, J. M., Photodegradation of avobenzone: Stabilization effect of antioxidants. *J Photochem Photobiol B Biol* **2014,** *140*, 36-40, 10.1016/j.jphotobiol.2014.07.004.
30. Tarras-Wahlberg, N.; Stenhagen, G.; Larko, O.; Rosen, A.; Wennberg, A. M.; Wennerstrom, O., Changes in ultraviolet absorption of sunscreens after ultraviolet irradiation. *J Invest Dermatol* **1999,** *113*, 547-553, 10.1046/j.1523-1747.1999.00721.x.
31. Schwack, W., Rudolph, T., Photochemistry of dibenzoyl methane uva filters - part 1. *J Photochem Photobiol B: Biol* **1995,** *28*, 229-234.
32. Downs, C. A.; DiNardo, J. C.; Stien, D.; Rodrigues, A. M. S.; Lebaron, P., Benzophenone accumulates over time from the degradation of octocrylene in commercial sunscreen products. *Chem Res Toxicol* **2021,** *34*, 1046-1054, 10.1021/acs.chemrestox.0c00461.
33. Roscher, N. M., Lindemann, M.K.O., Bin Kong, S., Cho, C.G., Jiang, P., Photodecomposition of several compounds commonly used as sunscreen agents. *J Photochem Photobiol A Chem* **1994,** *80*, 417-421.
34. Duarte, J.; Almeida, I. F.; Costa, M.; Da Silva, E. S.; Faria, J. L.; Sousa Lobo, J. M.; Costa, P. C.; Scalia, S., Alginate microparticles as carriers for the uv filter 2-ethylhexyl 4-methoxycinnamate: Influence on photostability. *Int J Cosmet Sci* **2019,** *41*, 585-593, 10.1111/ics.12578.
35. Chatelain, E.; Gabard, B., Photostabilization of butyl methoxydibenzoylmethane (avobenzone) and ethylhexyl methoxycinnamate by bis-ethylhexyloxyphenol methoxyphenyl triazine (tinosorb s), a new uv broadband filter. *Photochem Photobiol* **2001,** *74*, 401-406, 10.1562/0031-8655(2001)074<0401:POBMAA>2.0.CO;2.
36. Kockler, J.; Oelgemöller, M.; Robertson, S.; Glass, B. D., Influence of titanium dioxide particle size on the photostability of the chemical uv-filters butyl methoxy dibenzoylmethane and octocrylene in a microemulsion. *Cosmetics* **2014,** *1*, 128-139, 10.3390/cosmetics1020128.
37. Freitas, J. V.; Lopes, N. P.; Gaspar, L. R., Photostability evaluation of five uv-filters, trans-resveratrol and beta-carotene in sunscreens. *Eur J Pharm Sci* **2015,** *78*, 79-89, 10.1016/j.ejps.2015.07.004.
38. Karpkird, T.; Khunsakorn, R.; Noptheeranuphap, C.; Midpanon, S., Inclusion complexes and photostability of uv filters and curcumin with beta-cyclodextrin polymers: Effect on cross-linkers. *J Incl Phenom Macrocyclic Chem* **2018,** *91*, 37-45, 10.1007/s10847-018-0796-y.

39. Paris, C.; Lhiaubet-Vallet, V.; Jimenez, O.; Trullas, C.; Miranda, M. A., A blocked diketo form of avobenzone: Photostability, photosensitizing properties and triplet quenching by a triazine-derived uvb-filter. *Photochem Photobiol* **2009,** *85*, 178-184, 10.1111/j.1751-1097.2008.00414.x.

40. Coelho, L.; Almeida, I. F.; Sousa Lobo, J. M.; Sousa, E. S. J. P., Photostabilization strategies of photosensitive drugs. *Int J Pharm* **2018,** *541*, 19-25, 10.1016/j.ijpharm.2018.02.012.

41. de Oliveira, C. A.; Dario, M. F.; Sarruf, F. D.; Mariz, I. F. A.; Velasco, M. V. R.; Rosado, C.; Baby, A. R., Safety and efficacy evaluation of gelatin-based nanoparticles associated with uv filters. *Colloids Surf B Biointerfaces* **2016,** *140*, 531-537, 10.1016/j.colsurfb.2015.11.031.

42. Graziola, F.; Candido, T. M.; De Oliveira, C. A.; Peres, D. D.; Issa, M. G.; Mota, J.; Rosado, C.; Consiglieri, V. O.; Kaneko, T. M.; Velasco, M. V. R., *et al.*, Gelatin-based microspheres crosslinked with glutaraldehyde and rutin oriented to cosmetics. *Bras J Pharm Sci* **2016,** *52*, 603-612, 10.1590/S1984-82502016000400004.

43. Oliveira, C. A. D.; Peres, D. D.; Graziola, F.; Chacra, N. A. B.; Araújo, G. L. B. D.; Flórido, A. C.; Mota, J.; Rosado, C.; Velasco, M. V. R.; Rodrigues, L. M., *et al.*, Cutaneous biocompatible rutin-loaded gelatin-based nanoparticles increase the spf of the association of uva and uvb filters. *Eur J Pharm Sci* **2016,** *81*, 1-9, 10.1016/j.ejps.2015.09.016.

44. Al-Rawashdeh, N. A. F.; Al-Sadeh, K. S.; Al-Bitar, M. B., Physicochemical study on microencapsulation of hydroxypropyl-β-cyclodextrin in dermal preparations. *Drug Dev Ind Pharm* **2010,** *36*, 688-697, 10.3109/03639040903449738.

45. Scalia, S.; Casolari, A.; Iaconinoto, A.; Simeoni, S., Comparative studies of the influence of cyclodextrins on the stability of the sunscreen agent, 2-ethylhexyl-p-methoxycinnamate. *J Pharm Biomed Anal* **2002,** *30*, 1181-1189, 10.1016/S0731-7085(02)00433-8.

46. Scalia, S.; Tursilli, R.; Iannuccelli, V., Complexation of the sunscreen agent, 4-methylbenzylidene camphor with cyclodextrins: Effect on photostability and human stratum corneum penetration. *J Pharm Biomed Anal* **2007,** *44*, 29-34, 10.1016/j.jpba.2007.01.016.

47. Shokri, J.; Hasanzadeh, D.; Ghanbarzadeh, S.; Dizadji-Ilkhchi, M.; Adibkia, K., The effect of beta-cyclodextrin on percutaneous absorption of commonly used eusolex® sunscreens. *Drug Res* **2013,** *63*, 591-596, 10.1055/s-0033-1349089.

48. Gonzalez, H.; Tarras-Wahlberg, N.; Stromdahl, B.; Juzeniene, A.; Moan, J.; Larko, O.; Rosen, A.; Wennberg, A. M., Photostability of commercial sunscreens upon sun exposure and irradiation by ultraviolet lamps. *BMC Dermatol* **2007,** *7*, 1, 10.1186/1471-5945-7-1.

49. Shaath, N. A., Ultraviolet filters. *Photochem Photobiol Sci* **2010,** *9*, 464-469, 10.1039/b9pp00174c.

50. Huong, S. P.; Rocher, E. e.; Fourneron, J.-D.; Charles, L.; Monnier, V. e.; Bun, H.; Andrieu, V., Photoreactivity of the sunscreen butylmethoxydibenzoylmethane (dbm) under various experimental conditions. *J Photochem Photobiol A Chem* **2008,** *196*, 106-112, 10.1016/j.jphotochem.2007.11.023.

51. Juliano, C.; Magrini, G. A., Cosmetic ingredients as emerging pollutants of environmental and health concern. A mini-review. *Cosmetics* **2017,** *4*, 10.3390/cosmetics4020011.

52. Cunha, S. C.; Fernandes, J. O.; Vallecillos, L.; Cano-Sancho, G.; Domingo, J. L.; Pocurull, E.; Borrull, F.; Maulvault, A. L.; Ferrari, F.; Fernandez-Tejedor, M., *et al.*, Co-occurrence of musk fragrances and uv-filters in seafood and macroalgae collected in european hotspots. *Environ Res* **2015,** *143*, 65-71, 10.1016/j.envres.2015.05.003.

53. Gadelha, J. R.; Rocha, A. C.; Camacho, C.; Eljarrat, E.; Peris, A.; Aminot, Y.; Readman, J. W.; Boti, V.; Nannou, C.; Kapsi, M., *et al.*, Persistent and emerging pollutants assessment on aquaculture oysters (crassostrea gigas) from nw portuguese coast (ria de aveiro). *Sci Total Environ* **2019,** *666*, 731-742, 10.1016/j.scitotenv.2019.02.280.

54. Mitchelmore, C. L.; He, K.; Gonsior, M.; Hain, E.; Heyes, A.; Clark, C.; Younger, R.; Schmitt-Kopplin, P.; Feerick, A.; Conway, A., *et al.*, Occurrence and distribution of uv-filters and other anthropogenic contaminants in coastal surface water, sediment, and coral tissue from hawaii. *Sci Total Environ* **2019,** *670*, 398-410, 10.1016/j.scitotenv.2019.03.034.

55. Shick, J. M.; Lesser, M. P.; Jokiel, P. L., Effects of ultraviolet radiation on corals and other coral reef organisms. *Global Change Biol* **1996,** *2*, 527-545, 10.1111/j.1365-2486.1996.tb00065.x.

56. Wijgerde, T.; van Ballegooijen, M.; Nijland, R.; van der Loos, L.; Kwadijk, C.; Osinga, R.; Murk, A.; Slijkerman, D., Adding insult to injury: Effects of chronic oxybenzone exposure and elevated temperature on two reef-building corals. *Sci Total Environ* **2020,** *733*, 10.1016/j.scitotenv.2020.139030.
57. Corinaldesi, C.; Marcellini, F.; Nepote, E.; Damiani, E.; Danovaro, R., Impact of inorganic uv filters contained in sunscreen products on tropical stony corals (acropora spp.). *Sci Total Environ* **2018,** *637-638*, 1279-1285, 10.1016/j.scitotenv.2018.05.108.
58. Cahova, J.; Blahova, J.; Marsalek, P.; Doubkova, V.; Franc, A.; Garajová, M.; Tichy, F.; Mares, J.; Svobodova, Z., The biological activity of the organic uv filter ethylhexyl methoxycinnamate in rainbow trout (oncorhynchus mykiss). *Sci Total Environ* **2021,** *774*, 10.1016/j.scitotenv.2021.145570.
59. Gago-Ferrero, P.; Díaz-Cruz, M. S.; Barceló, D., Uv filters bioaccumulation in fish from iberian river basins. *Sci. Total Environ.* **2015,** *518-519*, 518-525, 10.1016/j.scitotenv.2015.03.026.
60. Balmer, M. E.; Buser, H. R.; Müller, M. D.; Poiger, T., Occurrence of some organic uv filters in wastewater, in surface waters, and in fish from swiss lakes. *Environ Sci Technol* **2005,** *39*, 953-962, 10.1021/es040055r.
61. Gago-Ferrero, P.; Alonso, M. B.; Bertozzi, C. P.; Marigo, J.; Barbosa, L.; Cremer, M.; Secchi, E. R.; Azevedo, A.; Lailson-Brito Jr, J.; Torres, J. P. M., *et al.*, First determination of uv filters in marine mammals. Octocrylene levels in franciscana dolphins. *Environ Sci Technol* **2013,** *47*, 5619-5625, 10.1021/es400675y.
62. Wawrzynczak, A.; Feliczak-Guzik, A.; Nowak, I., Nanosunscreens: From nanoencapsulated to nanosized cosmetic active forms. In *Nanobiomaterials in galenic formulations and cosmetics: Applications of nanobiomaterials*, Elsevier Inc.: 2016; pp 25-46.
63. Wang, S. Q., Balagula, Y., Osterwalder, U., Photoprotection: A review of the current and future technologies. *Dermatol Ther* **2010,** *23*, 31–47.
64. Janjua, N. R.; Mogensen, B.; Andersson, A. M.; Petersen, J. H.; Henriksen, M.; Skakkebaek, N. E.; Wulf, H. C., Systemic absorption of the sunscreens benzophenone-3, octyl-methoxycinnamate, and 3-(4-methyl-benzylidene) camphor after whole-body topical application and reproductive hormone levels in humans. *J Invest Dermatol* **2004,** *123*, 57-61, 10.1111/j.0022-202X.2004.22725.x.
65. Rodriguez, I., Fenollosa, R.,, Meseguer, F. Silicon microspheres as uv, visible and infrared filters for cosmetics. https://www.cosmeticsandtoiletries.com/formulating/function/uvfilter/premium-Silicon-Microspheres-as-UV-Visible-and-Infrared-Filters-for-Cosmetics-208701831.html (12th July),
66. Ambrogi, V.; Latterini, L.; Marmottini, F.; Pagano, C.; Ricci, M., Mesoporous silicate mcm-41 as a particulate carrier for octyl methoxycinnamate: Sunscreen release and photostability. *J Pharm Sci* **2013,** *102*, 1468-1475, 10.1002/jps.23478.
67. Revised guidance document 150 on standardised test guidelines for evaluating chemicals for endocrine disruption. Development, O.-O. f. E. C.-o. a., Ed. OECD Publishing, Paris: 2018.
68. Witorsch, R. J.; Thomas, J. A., Personal care products and endocrine disruption: A critical review of the literature. *Crit Rev Toxicol* **2010,** *40*, 1-30, 10.3109/10408444.2010.515563.
69. Rehfeld, A.; Dissing, S.; Skakkebæk, N. E., Chemical uv filters mimic the effect of progesterone on ca2+ signaling in human sperm cells. *Endocrinology* **2016,** *157*, 4297-4308, 10.1210/en.2016-1473.
70. Ponzo, O. J.; Silvia, C., Evidence of reproductive disruption associated with neuroendocrine changes induced by uv-b filters, phtalates and nonylphenol during sexual maturation in rats of both gender. *Toxicology* **2013,** *311*, 41-51, 10.1016/j.tox.2013.05.014.
71. Axelstad, M.; Boberg, J.; Hougaard, K. S.; Christiansen, S.; Jacobsen, P. R.; Mandrup, K. R.; Nellemann, C.; Lund, S. P.; Hass, U., Effects of pre- and postnatal exposure to the uv-filter octyl methoxycinnamate (omc) on the reproductive, auditory and neurological development of rat offspring. *Toxicol Appl Pharmacol* **2011,** *250*, 278-290, 10.1016/j.taap.2010.10.031.
72. Ozáez, I.; Martínez-Guitarte, J. L.; Morcillo, G., Effects of in vivo exposure to uv filters (4-mbc, omc, bp-3, 4-hb, oc, od-paba) on endocrine signaling genes in the insect chironomus riparius. *Sci Total Environ* **2013,** *456-457*, 120-126, 10.1016/j.scitotenv.2013.03.081.

73. Li, A. J.; Law, J. C. F.; Chow, C. H.; Huang, Y.; Li, K.; Leung, K. S. Y., Joint effects of multiple uv filters on zebrafish embryo development. *Environ Sci Technol* **2018,** *52*, 9460-9467, 10.1021/acs.est.8b02418.

74. Li, V. W. T.; Tsui, M. P. M.; Chen, X.; Hui, M. N. Y.; Jin, L.; Lam, R. H. W.; Yu, R. M. K.; Murphy, M. B.; Cheng, J.; Lam, P. K. S., *et al.*, Effects of 4-methylbenzylidene camphor (4-mbc) on neuronal and muscular development in zebrafish (danio rerio) embryos. *Environ Sci Pollut Res* **2016,** *23*, 8275-8285, 10.1007/s11356-016-6180-9.

75. Lorigo, M.; Mariana, M.; Cairrao, E., Photoprotection of ultraviolet-b filters: Updated review of endocrine disrupting properties. *Steroids* **2018,** *131*, 46-58, 10.1016/j.steroids.2018.01.006.

76. Balázs, A.; Krifaton, C.; Orosz, I.; Szoboszlay, S.; Kovács, R.; Csenki, Z.; Urbányi, B.; Kriszt, B., Hormonal activity, cytotoxicity and developmental toxicity of uv filters. *Ecotoxicol Environ Saf* **2016,** *131*, 45-53, 10.1016/j.ecoenv.2016.04.037.

77. Chen, T. H.; Hsieh, C. Y.; Ko, F. C.; Cheng, J. O., Effect of the uv-filter benzophenone-3 on intra-colonial social behaviors of the false clown anemonefish (amphiprion ocellaris). *Sci Total Environ* **2018,** *644*, 1625-1629, 10.1016/j.scitotenv.2018.07.203.

78. Carvalhais, A.; Pereira, B.; Sabato, M.; Seixas, R.; Dolbeth, M.; Marques, A.; Guilherme, S.; Pereira, P.; Pacheco, M.; Mieiro, C., Mild effects of sunscreen agents on a marine flatfish: Oxidative stress, energetic profiles, neurotoxicity and behaviour in response to titanium dioxide nanoparticles and oxybenzone. *Int J Mol Sci* **2021,** *22*, 1-25, 10.3390/ijms22041567.

79. Pablos, M. V.; Garcia-Hortiguela, P.; Fernandez, C., Acute and chronic toxicity of emerging contaminants, alone or in combination, in chlorella vulgaris and daphnia magna. *Environ Sci Pollut Res Int* **2015,** *22*, 5417-5424, 10.1007/s11356-015-4119-1.

80. Elder, D. P.; Snodin, D. J., Drug substances presented as sulfonic acid salts: Overview of utility, safety and regulation. *J Pharm Pharmacol* **2009,** *61*, 269-278, 10.1211/jpp/61.03.0001.

81. Lozano, C.; Matallana-Surget, S.; Givens, J.; Nouet, S.; Arbuckle, L.; Lambert, Z.; Lebaron, P., Toxicity of uv filters on marine bacteria: Combined effects with damaging solar radiation. *Sci Total Environ* **2020,** *722*, 10.1016/j.scitotenv.2020.137803.

82. Huang, Y.; Law, J. C. F.; Lam, T. K.; Leung, K. S. Y., Risks of organic uv filters: A review of environmental and human health concern studies. *Sci Total Environ* **2021,** *755*, 10.1016/j.scitotenv.2020.142486.

83. Pico, Y.; Belenguer, V.; Corcellas, C.; Diaz-Cruz, M. S.; Eljarrat, E.; Farre, M.; Gago-Ferrero, P.; Huerta, B.; Navarro-Ortega, A.; Petrovic, M., *et al.*, Contaminants of emerging concern in freshwater fish from four spanish rivers. *Sci Total Environ* **2019,** *659*, 1186-1198, 10.1016/j.scitotenv.2018.12.366.

84. Tsui, M. M. P.; Lam, J. C. W.; Ng, T. Y.; Ang, P. O.; Murphy, M. B.; Lam, P. K. S., Occurrence, distribution, and fate of organic uv filters in coral communities. *Environ Sci Technol* **2017,** *51*, 4182-4190, 10.1021/acs.est.6b05211.

85. The Senate, T.-n. L., State of Hawaii. Https://www.Capitol.Hawaii.Gov/session2018/bills/sb2571_.Htm. (29th April 2021),

86. Danovaro, R.; Bongiorni, L.; Corinaldesi, C.; Giovannelli, D.; Damiani, E.; Astolfi, P.; Greci, L.; Pusceddu, A., Sunscreens cause coral bleaching by promoting viral infections. *Environ Health Perspect* **2008,** *116*, 441-447, 10.1289/ehp.10966.

87. Picot Groz, M.; Martinez Bueno, M. J.; Rosain, D.; Fenet, H.; Casellas, C.; Pereira, C.; Maria, V.; Bebianno, M. J.; Gomez, E., Detection of emerging contaminants (uv filters, uv stabilizers and musks) in marine mussels from portuguese coast by quechers extraction and gc-ms/ms. *Sci Total Environ* **2014,** *493*, 162-169, 10.1016/j.scitotenv.2014.05.062.

88. Thorel, E.; Clergeaud, F.; Jaugeon, L.; Rodrigues, A. M. S.; Lucas, J.; Stien, D.; Lebaron, P., Effect of 10 uv filters on the brine shrimp artemia salina and themarinemicroalga tetraselmis sp. *Toxics* **2020,** *8*, 10.3390/TOXICS8020029.

89. Mao, F.; He, Y.; Gin, K. H., Evaluating the joint toxicity of two benzophenone-type uv filters on the green alga chlamydomonas reinhardtii with response surface methodology. *Toxics* **2018,** *6*, 10.3390/toxics6010008.

90. Boyd, A.; Stewart, C. B.; Philibert, D. A.; How, Z. T.; El-Din, M. G.; Tierney, K. B.; Blewett, T. A., A burning issue: The effect of organic ultraviolet filter exposure on the behaviour and physiology of daphnia magna. *Sci Total Environ* **2021,** *750*, 10.1016/j.scitotenv.2020.141707.

91. Quintaneiro, C.; Teixeira, B.; Benedé, J. L.; Chisvert, A.; Soares, A. M. V. M.; Monteiro, M. S., Toxicity effects of the organic uv-filter 4-methylbenzylidene camphor in zebrafish embryos. *Chemosphere* **2019,** *218*, 273-281, 10.1016/j.chemosphere.2018.11.096.

92. Pawlowski, S.; Herzog, B.; Sohn, M.; Petersen-Thiery, M.; Acker, S., Ecosun pass: A tool to evaluate the ecofriendliness of uv filters used in sunscreen products. *Int J Cosmet Sci* **2021,** *43*, 201-210, 10.1111/ics.12681.

93. Bordalo, D.; Leite, C.; Almeida, Â.; Soares, A. M. V. M.; Pretti, C.; Freitas, R., Impacts of uv filters in mytilus galloprovincialis: Preliminary data on the acute effects induced by environmentally relevant concentrations. *Sustainability* **2020,** *12*, 10.3390/SU12176852.

94. Krutmann, J.; Passeron, T.; Gilaberte, Y.; Granger, C.; Leone, G.; Narda, M.; Schalka, S.; Trullas, C.; Masson, P.; Lim, H. W., Photoprotection of the future: Challenges and opportunities. *J Eur Acad Dermatol Venereol* **2020,** *34*, 447-454, 10.1111/jdv.16030.

95. Yarovaya, L.; Waranuch, N.; Wisuitiprot, W.; Khunkitti, W., Effect of grape seed extract on skin fibroblasts exposed to uva light and its photostability in sunscreen formulation. *J Cosmet Dermatol* **2020**, 10.1111/jocd.13711.

96. Hubner, A.; Sobreira, F.; Neto, A. V.; de Oliveira Pinto, C. A. S.; Dario, M. F.; Díaz, I. E. C.; Lourenço, F. R.; Rosado, C.; Baby, A. R.; Bacchi, E. M., The synergistic behavior of antioxidant phenolic compounds obtained fromwinemaking waste's valorization, increased the efficacy of a sunscreen system. *Antioxidants* **2019,** *8*, 10.3390/antiox8110530.

97. da Silva, A. C. P.; Paiva, J. P.; Diniz, R. R.; dos Anjos, V. M.; Silva, A. B. S. M.; Pinto, A. V.; dos Santos, E. P.; Leitão, A. C.; Cabral, L. M.; Rodrigues, C. R., *et al.*, Photoprotection assessment of olive (olea europaea l.) leaves extract standardized to oleuropein: In vitro and in silico approach for improved sunscreens. *J Photochem Photobiol B Biol* **2019,** *193*, 162-171, 10.1016/j.jphotobiol.2019.03.003.

98. Polonini, H. C.; Brandão, M. A. F.; Raposo, N. R. B., A natural broad-spectrum sunscreen formulated from the dried extract of brazilian lippia sericea as a single uv filter. *RSC Adv* **2014,** *4*, 62566-62575, 10.1039/c4ra11577e.

99. Alves, G. D. A. D.; de Souza, R. O.; Rogez, H.; Masaki, H.; Fonseca, M. J. V., Cecropia obtusa, an amazonian ethanolic extract, exhibits photochemoprotective effect in vitro and balances the redox cellular state in response to uv radiation. *Ind Crops Prod* **2016,** *94*, 893-902, 10.1016/j.indcrop.2016.09.064.

100. Anurukvorakun, O.; Boonruang, R.; Lahpun, N., Formulation strategy, stability issues, safety and efficacy evaluations of acacia catechu whitening cream. *Int J Appl Pharm* **2019,** *11*, 91-96, 10.22159/ijap.2019v11i2.30632.

101. Wróblewska, K. B.; Baby, A. R.; Grombone Guaratini, M. T.; Moreno, P. R. H., In vitro antioxidant and photoprotective activity of five native brazilian bamboo species. *Ind Crops Prod* **2019,** *130*, 208-215, 10.1016/j.indcrop.2018.12.081.

102. Lohezic-Le Devehat, F.; Legouin, B.; Couteau, C.; Boustie, J.; Coiffard, L., Lichenic extracts and metabolites as uv filters. *J Photochem Photobiol B Biol* **2013,** *120*, 17-28, 10.1016/j.jphotobiol.2013.01.009.

103. Lee, S. C.; Lee, S. H.; Won, K., Wood powder as a new natural sunscreen ingredient. *Biotechnol. Bioprocess Eng.* **2019,** *24*, 258-263, 10.1007/s12257-018-0397-z.

104. Rasheed, A.; Shama, S. N.; Mohanalakshmi, S.; Ravichandran, V., Formulation, characterization and in vitro evaluation of herbal sunscreen lotion. *Orient Pharm Exp Med* **2012,** *12*, 241-246, 10.1007/s13596-012-0069-z.

105. Widiyati, E. In *Determination of ultraviolet filter activity on coconut oil cosmetic cream*, Suyanto, S.; Kusumawardani, C.; Wilujeng, I.; Abadi, A. M., Eds. American Institute of Physics Inc.: 2017.

106. Bhattacharya, S.; Sherje, A. P., Development of resveratrol and green tea sunscreen formulation for combined photoprotective and antioxidant properties. *J Drug Deliv Sci Technol* **2020,** *60*, 10.1016/j.jddst.2020.102000.

107. L'Alloret, F.; Candau, D.; Seité, S.; Pygmalion, M. J.; Ruiz, L.; Josso, M.; Meaudre, H.; Gauchet, L.; Pena, A. M.; Colonna, A., New combination of ultraviolet absorbers in an oily emollient increases sunscreen efficacy and photostability. *Dermatol Ther* **2012,** *2*, 1-10, 10.1007/s13555-012-0004-8.

108. Montenegro, L.; Santagati, L. M., Use of vegetable oils to improve the sun protection factor of sunscreen formulations. *Cosmetics* **2019,** *6*, 10.3390/COSMETICS6020025.

109. Mota, M. D.; Costa, R. Y. S.; Guedes, A. A. S.; Silva, L. C. R. C. E.; Chinalia, F. A., Guava-fruit extract can improve the uv-protection efficiency of synthetic filters in sun cream formulations. *J Photochem Photobiol B Biol* **2019,** *201*, 10.1016/j.jphotobiol.2019.111639.

110. Milani, L. P. G.; Garcia, N. O. S.; Morais, M. C.; Dias, A. L. S.; Oliveira, N. L.; Conceição, E. C., Extract from byproduct psidium guajava standardized in ellagic acid: Additivation of the in vitro photoprotective efficacy of a cosmetic formulation. *Rev Bras Farmacogn* **2018,** *28*, 692-696, 10.1016/j.bjp.2018.08.005.

111. Niculae, G.; Lacatusu, I.; Badea, N.; Stan, R.; Vasile, B. S.; Meghea, A., Rice bran and raspberry seed oil-based nanocarriers with self-antioxidative properties as safe photoprotective formulations. *Photochem Photobiol Sci* **2014,** *13*, 703-716, 10.1039/c3pp50290b.

112. Almeida, W. A. D. S.; Antunes, A. D. S.; Penido, R. G.; Correa, H. S. D. G.; Nascimento, A. M. D.; Andrade, Â. L.; Santos, V. R.; Cazati, T.; Amparo, T. R.; Souza, G. H. B. D., *et al.*, Photoprotective activity and increase of spf in sunscreen formulation using lyophilized red propolis extracts from alagoas. *Rev Bras Farmacogn* **2019,** *29*, 373-380, 10.1016/j.bjp.2019.02.003.

113. Llewellyn, C. A.; Airs, R. L., Distribution and abundance of maas in 33 species of microalgae across 13 classes. *Mar Drugs* **2010,** *8*, 1273-1291, 10.3390/md8041273.

114. Liu, Z., Occurrence of mycosporine-like amino acids (maas) in the bloom-forming cyanobacterium microcystis aeruginosa. *J Plankton Res* **2004,** *26*, 963-966, 10.1093/plankt/fbh083.

115. Rangel, K. C.; Villela, L. Z.; Pereira, K. D. C.; Colepicolo, P.; Debonsi, H. M.; Gaspar, L. R., Assessment of the photoprotective potential and toxicity of antarctic red macroalgae extracts from curdiea racovitzae and iridaea cordata for cosmetic use. *Algal Res* **2020,** *50*, 10.1016/j.algal.2020.101984.

116. Prasedya, E. S.; Syafitri, S. M.; Geraldine, B. A. F. D.; Hamdin, C. D.; Frediansyah, A.; Miyake, M.; Kobayashi, D.; Hazama, A.; Sunarpi, H., Uva photoprotective activity of brown macroalgae sargassum cristafolium. *Biomedicines* **2019,** *7*, 10.3390/biomedicines7040077.

117. Proteau, P. J., Gerwick, W. H., Garcia-Pichel , F., Castenholz, R., The structure of scytonemin, an ultraviolet sunscreen pigment from the sheaths of cyanobacteria. *Experientia* **1993,** *49*, 825-829.

118. Kang, M. R.; Jo, S. A.; Lee, H.; Yoon, Y. D.; Kwon, J. H.; Yang, J. W.; Choi, B. J.; Park, K. H.; Lee, M. Y.; Lee, C. W., *et al.*, Inhibition of skin inflammation by scytonemin, an ultraviolet sunscreen pigment. *Mar Drugs* **2020,** *18*, 10.3390/md18060300.

119. Teixeira, T. R.; Rangel, K. C.; Tavares, R. S. N.; Kawakami, C. M.; Dos Santos, G. S.; Maria-Engler, S. S.; Colepicolo, P.; Gaspar, L. R.; Debonsi, H. M., In vitro evaluation of the photoprotective potential of quinolinic alkaloids isolated from the antarctic marine fungus penicillium echinulatum for topical use. *Marine Biotechnol (NY)* **2021**, 10.1007/s10126-021-10030-x.

120. Maciel, O. M. C.; Tavares, R. S. N.; Caluz, D. R. E.; Gaspar, L. R.; Debonsi, H. M., Photoprotective potential of metabolites isolated from algae-associated fungi annulohypoxylon stygium. *J Photochem Photobiol B Biol* **2018,** *178*, 316-322, 10.1016/j.jphotobiol.2017.11.018.

121. Regalado, E. L.; Rodriguez, M.; Menendez, R.; Concepcion, A. A.; Nogueiras, C.; Laguna, A.; Rodriguez, A. A.; Williams, D. E.; Lorenzo-Luaces, P.; Valdes, O., *et al.*, Repair of uvb-damaged skin by the antioxidant sulphated flavone glycoside thalassiolin b isolated from the marine plant thalassia testudinum banks ex konig. *Marine Biotechnol (NY)* **2009,** *11*, 74-80, 10.1007/s10126-008-9123-8.

122. Ahmed, F. E., Setlow, R. B., Ultraviolet radiation-induced DNA damage and its photorepair in the skin of the platyfish xiphophorus. *Cancer Res* **1993,** *53*, 2249-2255.

123. Ryu, B., Qian, Z.-J., Kim, M.-M., Nam, K. W., Kim, S.-K., Anti-photoaging activity and inhibition of matrix metalloproteinase (mmp) by marine red alga, corallina pilulifera methanol extract. *Radiat Phys Chem* **2009,** *78,* 98-105, 10.1016/j.radphyschem.2008.09.001.

124. Yabe, S.; Sato, T., Cerium oxide for sunscreen cosmetics. *J Solid State Chem* **2003,** *171*, 7-11, 10.1016/S0022-4596(02)00139-1.

125. Seixas, V. C.; Serra, O. A., Stability of sunscreens containing cepo4: Proposal for a new inorganic uv filter. *Molecules* **2014,** *19*, 9907-9925, 10.3390/molecules19079907.
126. Polonini, H. C.; Lopes, R. S.; Beatriz, A.; Gomes, R. S.; Silva, A. O.; De Lima, R. V.; Nunes, G. A.; Brandão, M. A. F.; Raposo, N. R. B.; De Lima, D. P., Synthesis and evaluation of octocrylene-inspired compounds for uv-filter activity. *Quim Nova* **2014,** *37*, 1004-1009, 10.5935/0100-4042.20140160.
127. González, M. T. P.; Fumagalli, F.; Benevenuto, C. G.; da Silva Emery, F.; Gaspar, L. R., Novel benzophenone-3 derivatives with promising potential as uv filters: Relationship between structure, photoprotective potential and phototoxicity. *Eur J Pharm Sci* **2017,** *101*, 200-210, 10.1016/j.ejps.2017.02.014.
128. Gonçalves, M. C.; Rossoni, J. V., Jr.; Rabelo, A. C. S.; Costa, D. C.; Cazati, T.; Taylor, J. G.; Dos Santos, V. M. R., Preliminary studies of the cytotoxicity and photoprotective properties of benzophenone and lactone derivatives. *Rev. Virtual Quim* **2018,** *10*, 600-608, 10.21577/1984-6835.20180044.
129. Abbas, N.; Mahmood, Z.; Manzoor, S.; Akhtar, Z.; Tariq, M., Synthesis, characterization and sunscreen protection factor of novel synthesized para amino benzoic acid (paba) esters. *J Chem Soc Pak* **2019,** *41*, 701-706.
130. Bino, A.; Baldisserotto, A.; Scalambra, E.; Dissette, V.; Vedaldi, D. E.; Salvador, A.; Durini, E.; Manfredini, S.; Vertuani, S., Design, synthesis and biological evaluation of novel hydroxy-phenyl-1h-benzimidazoles as radical scavengers and uv-protective agents. *J Enzyme Inhib Med Chem* **2017,** *32*, 527-537, 10.1080/14756366.2016.1265523.
131. Djuidje, E. N.; Durini, E.; Sciabica, S.; Serra, E.; Balzarini, J.; Liekens, S.; Manfredini, S.; Vertuani, S.; Baldisserotto, A., Skin damages—structure activity relationship of benzimidazole derivatives bearing a 5-membered ring system. *Molecules* **2020,** *25*, 10.3390/molecules25184324.
132. Popiół, J.; Gunia-Krzyzak, A.; Piska, K.; Zelaszczyk, D.; Koczurkiewicz, P.; Słoczynska, K.; Wójcik-Pszczoła, K.; Krupa, A.; Kryczyk-Poprawa, A.; Zesławska, E., *et al.*, Discovery of novel uv-filters with favorable safety profiles in the 5-arylideneimidazolidine-2,4-dione derivatives group. *Molecules* **2019,** *24*, 10.3390/molecules24122321.
133. Peyrot, C.; Mention, M. M.; Brunissen, F.; Allais, F., Sinapic acid esters: Octinoxate substitutes combining suitable uv protection and antioxidant activity. *Antioxidants* **2020,** *9*, 1-16, 10.3390/antiox9090782.
134. Reis, J. S.; Corrêa, M. A.; Chung, M. C.; Dos Santos, J. L., Synthesis, antioxidant and photoprotection activities of hybrid derivatives useful to prevent skin cancer. *Bioorg Med Chem* **2014,** *22*, 2733-2738, 10.1016/j.bmc.2014.03.017.
135. Polonini, H. C.; Lima, L. L.; Gonçalves, K. M.; Do Carmo, A. M. R.; Da Silva, A. D.; Raposo, N. R. B., Photoprotective activity of resveratrol analogues. *Bioorg Med Chem* **2013,** *21*, 964-968, 10.1016/j.bmc.2012.11.052.
136. Horbury, M. D.; Holt, E. L.; Mouterde, L. M. M.; Balaguer, P.; Cebrian, J.; Blasco, L.; Allais, F.; Stavros, V. G., Towards symmetry driven and nature inspired uv filter design. *Nat Commun* **2019,** *10*, 4748, 10.1038/s41467-019-12719-z.
137. Rioux, B.; Peyrot, C.; Mention, M. M.; Brunissen, F.; Allais, F., Sustainable synthesis of p-hydroxycinnamic diacids through proline-mediated knoevenagel condensation in ethanol: An access to potent phenolic uv filters and radical scavengers. *Antioxidants* **2020,** *9*, 10.3390/antiox9040331.
138. Resende, D. I. S. P.; Almeida, M. C.; Maciel, B.; Carmo, H.; Lobo, J. S.; Pozzo, C. D.; Cravo, S. M.; Rosa, G. P.; Kane-Pagès, A.; Do Carmo Barreto, M., *et al.*, Efficacy, stability, and safety evaluation of new polyphenolic xanthones towards identification of bioactive compounds to fight skin photoaging. *Molecules* **2020,** *25*, 10.3390/molecules25122782.
139. Polonini, H. C.; Dias, R. M. P.; Souza, I. O.; Gonçalves, K. M.; Gomes, T. B. B.; Raposo, N. R. B.; Da Silva, A. D., Quinolines derivatives as novel sunscreening agents. *Bioorg Med Chem Lett* **2013,** *23*, 4506-4510, 10.1016/j.bmcl.2013.06.046.
140. Giacomo, B.; Luca, B.; Nicola, L.; Luigi, R., Development of an innovative and eco-friendly uv radiation absorber, based on furan moieties. *Cosmetics* **2020,** *7*, 10.3390/cosmetics7010006.
141. Peyrot, C.; Mention, M. M.; Brunissen, F.; Balaguer, P.; Allais, F., Innovative bio-based organic uv-a and blue light filters from meldrum's acid. *Molecules* **2020,** *25*, 10.3390/molecules25092178.
